# Mitochondrial quality control: a pathophysiological mechanism and potential therapeutic target for chronic obstructive pulmonary disease

**DOI:** 10.3389/fphar.2024.1474310

**Published:** 2025-01-03

**Authors:** Mengjiao Xu, Peng Feng, Jun Yan, Lei Li

**Affiliations:** ^1^ Wangjing Hospital, China Academy of Chinese Medical Sciences, Beijing, China; ^2^ Ferguson Laboratory, Department of Orthopaedic Surgery, University of Pittsburgh, Pittsburgh, PA, United States; ^3^ Dongzhimen Hospital, Beijing University of Chinese Medicine, Beijing, China

**Keywords:** chronic obstructive pulmonary disease, mitochondrial dysfunction, mitochondrial quality control, mitophagy, mitochondrial dynamics, mitochondrial biogenesis, therapeutic strategies

## Abstract

Chronic obstructive pulmonary disease (COPD) is a prevalent chronic respiratory disease worldwide. Mitochondrial quality control mechanisms encompass processes such as mitochondrial biogenesis, fusion, fission, and autophagy, which collectively maintain the quantity, morphology, and function of mitochondria, ensuring cellular energy supply and the progression of normal physiological activities. However, in COPD, due to the persistent stimulation of harmful factors such as smoking and air pollution, mitochondrial quality control mechanisms often become deregulated, leading to mitochondrial dysfunction. Mitochondrial dysfunction plays a pivotal role in the pathogenesis of COPD, contributing toinflammatory response, oxidative stress, cellular senescence. However, therapeutic strategies targeting mitochondria remain underexplored. This review highlights recent advances in mitochondrial dysfunction in COPD, focusing on the role of mitochondrial quality control mechanisms and their dysregulation in disease progression. We emphasize the significance of mitochondria in the pathophysiological processes of COPD and explore potential strategies to regulate mitochondrial quality and improve mitochondrial function through mitochondrial interventions, aiming to treat COPD effectively. Additionally, we analyze the limitations and challenges of existing therapeutic strategies, aiming to provide new insights and methods for COPD treatment.

## 1 Introduction

Mitochondria are key organelles in eukaryotic cells that play a central role in cellular energy metabolism. As the “power plant” of the cell, mitochondria generate ATP through oxidative phosphorylation (OXPHOS) to provide necessary energy for cells and actively participate in a variety of biological processes, such as apoptosis, calcium homeostasis, reactive oxygen species (ROS) generation and lipid metabolism (Eshraghi et al., 2021; Murphy et al., 2016). Their highly dynamic double-membrane structure and complex internal biochemical reactions ensure an efficient energy conversion and regulation centre and play a key role in maintaining cellular homeostasis (Li A. et al., 2020). To safeguard normal mitochondrial functioning, cells have developed complex mitochondrial quality control mechanisms, mainly involving mitophagy, mitochondrial dynamics, and mitochondrial biogenesis, effectively monitoring and maintaining the integrity of the intracellular mitochondrial network by regulating the number, morphology, and function of mitochondria, ensuring stable mitochondrial function and cellular health (An et al., 2021). Additionally, the regulation of mitochondrial ROS (mtROS), repair of mtDNA, UPRmt, and contact between mitochondria and other organelles, such as the endoplasmic reticulum, are part of the mitochondrial quality control system (Ng et al., 2021).

Chronic obstructive pulmonary disease (COPD) is a progressive chronic progressive respiratory disease characterised by persistent airway inflammation and incomplete reversible airflow obstruction mainly due to long-term smoking or exposure to harmful environmental factors, such as occupational dust and air pollution. By 2030, COPD is projected to be the third leading cause of death worldwide, posing a major public health challenge (Fazleen and Wilkinson, 2020). Low rates of awareness and diagnosis put patients at high risk of acute exacerbation, moderate-to-severe airflow obstruction, and various complications, increasing disability and mortality rates and causing a substantial disease burden on society. Despite various treatment strategies, COPD remains difficult to reverse, and the risk of acute exacerbation in patients remains high (Agustí and Hogg, 2019; Ritchie and Wedzicha, 2020). Recently, the role of mitochondria in COPD has received widespread attention, and mitochondrial dysfunction has been regarded as a key link in the pathogenesis of COPD. A recent study showed that in a COPD cell model, stimulation of 16 Human Bronchial Epithelial (16HBE) Cells with BRPM2.5 resulted in a substantial increase in cell ROS and mtROS; excessive ROS caused reduced ΔΨm and ATP levels, and impaired mitochondrial function with altered mitochondrial dynamics, exacerbating inflammatory response and promoting COPD progression (Gao et al., 2022).

Mitochondrial quality control is a regulatory mechanism for the bioenergetic changes that occur in response to mitochondrial dysfunction (Larson-Casey et al., 2020). When cells are subjected to inflammatory stimuli, oxidative stress, or external physical factors that impair mitochondrial function, mitochondrial quality control is rapidly activated in response to this challenge (Boyman et al., 2020). Mitophagy is crucial in clearing damaged or dysfunctional mitochondria,preventing further cell damage (Campos et al., 2016; Springer and Macleod, 2016). Mitochondrial dynamics maintains the mitochondrial morphological and functional homeostasis by regulating the dynamic balance between mitochondrial fusion and fission (Wai and Langer, 2016). Additionally, mitochondrial biogenesis ensures the energy supply for cell growth and repair. These processes collaborate to fulfill the mitochondrial energy demand of cells and safeguard cellular health. In one study peroxisome proliferator-activated receptor gamma coactivator 1-alpha (PGC1-α) expression levels showed dynamic changes during the development of COPD. (Li et al., 2010). In the lung tissue of patients with mild COPD, PGC1-α level was increased, which may be a compensatory mechanism that attempted to respond to initial oxidative stress and inflammatory damage by enhancing mitochondrial biogenesis; however, as the disease progresses to moderate-severe stages, PGC1-α levels progressively decreased, indicating that the mitochondrial biogenesis process was severely impaired (Li et al., 2010). When mitochondrial damage exceeds the clearance capacity of the quality control mechanisms, a disordered state that fails to effectively deal with mitochondrial dysfunction ensues, further exacerbating functional abnormalities and creating a vicious cycle that may contribute to COPD progression. Therefore, therapeutic strategies targeting mitochondrial quality control are essential to break this cycle and improve COPD outcomes.

This review will explore how mitochondrial quality control contributes to the pathogenesis of COPD, and evaluate the potential application of therapeutic strategies targeting mitochondrial quality control in COPD treatment, aiming to provide new insights for basic research and clinical treatment of COPD.

## 2 Methods

### 2.1 Literature search

PubMed, Web of Science, China National Knowledge Infrastructure (CNKI) were searched, using keywords such as “Chronic obstructive pulmonary disease”, “Mitochondrial dysfunction”, “Mitochondrial quality control”, “Mitophagy”, “Mitochondrial dynamics”, “Mitochondrial biogenesis”, “Therapeutic strategies” to retrieve studies in ameliorating COPD by targeting mitochondria. The search period was from database inception to May 2024.

### 2.2 Inclusion criteria

Studies on the treatment of COPD by targeting mitochondria, encompassing the modulation of mtROS, mitophagy, mitochondrial dynamics, and mitochondrial biogenesis.

### 2.3 Exclusion criteria

Duplicate and incomplete studies, conference abstracts, and studies lacking ethical approval were excluded.

### 2.4 Results

**FIGURE 1:**
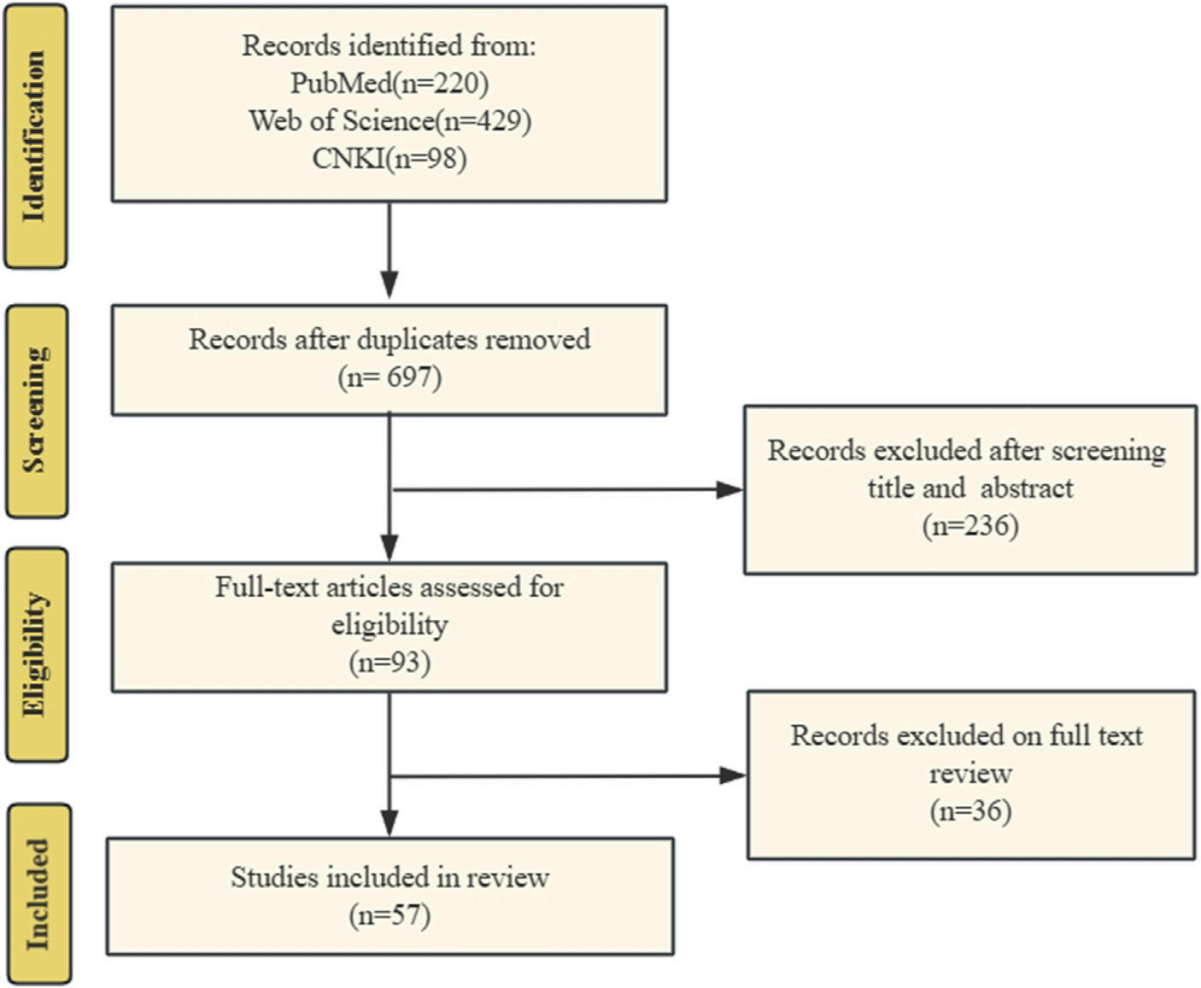
The literature search and screening flowchart. We searched PubMed, Web of Science, and CNKI for literature on targeted mitochondrial quality control for COPD treatment. Search history following the PRISMA criteria.

Two researchers independently conducted searches using relevant keywords and subsequently screened the titles, abstracts, and full texts individually, adhering to both inclusion and exclusion criteria, ultimately resulting in the selection of 57 eligible publications. The literature search and screening flowchart is shown in Figure 1.

## 3 Mitochondrial dysfunction

Mitochondrial dysfunction is closely associated with various factors, including oxidative stress, mtDNA damage, metal ion homeostasis, mitophagy, apoptosis, metabolic disorders, aging, and environmental toxins and drugs. Mitochondrial dysfunction significantly impacts the progression and outcomes of various diseases. Impaired mitochondria result in excessive ROS production on the respiratory chain, leading to oxidative damage to cellular and mitochondrial proteins, lipids, and DNA (Tang et al., 2021; Dubois-Deruy et al., 2020). This oxidative damage can further exacerbate mitochondrial dysfunction, creating a vicious cycle. Key manifestations of mitochondrial dysfunction include excessive mtROS accumulation, mtDNA damage and mutations, reduced ATP synthesis, and altered mitochondrial membrane potential (MMP). Excessive mtROS production and mtDNA damage and mutations are critical markers of mitochondrial dysfunction and pivotal factors in mitochondrial quality control disruption. mtROS, a byproduct of the mitochondrial respiratory chain, normally plays a significant role in cellular signaling and homeostasis. However, in COPD, increased oxidative stress leads to excessive mtROS production, inducing oxidative stress, abnormal mitophagy, and mitochondrial dysfunction (Fan et al., 2023). mtDNA is the genetic material in mitochondria, responsible for encoding proteins related to energy production and other crucial mitochondrial functions. Tobacco smoke exposure induces mtDNA mutations and damage, triggering mitochondrial dysfunction and exacerbating COPD progression (Giordano et al., 2022). In the pathogenesis of COPD, disrupted mitochondrial function, exacerbated by dysfunctional mitochondrial quality control mechanisms, forms a vicious cycle.

### 3.1 Free-radical generation and oxidative damage in COPD

Abnormal accumulation of mtROS is a hallmark of mitochondrial dysfunction. mtROS production is influenced by the redox states of Nicotinamide Adenine Dinucleotide (NAD) and Flavin Adenine Dinucleotide (FAD) pools, oxygen availability, mitochondrial structure, and the insulation of electron carriers in the inner membrane (Palma et al., 2024). Mitochondria generate ATP via OXPHOS, during which electrons are transferred from complexes I or II through complex III and then to complex IV of the electron transport chain (ETC), ultimately reacting with oxygen to form water. In addition to these complexes, other electron carriers like coenzyme Q10 (CoQ10) and Cytochrome c Oxidase (cytochrome c) also facilitate this transfer (Palma et al., 2024). Proton pumping occurs primarily at complexes I, III, and IV, moving protons from the mitochondrial matrix to the intermembrane space. Electron leakage occurs when NAD⁺ and FAD become overly reduced or when quinone is partially reduced to semiquinone, increasing the likelihood of superoxide or hydrogen peroxide formation within mitochondria.

Moderate levels of mtROS act as cellular signaling molecules, regulating various cellular functions and maintaining physiological homeostasis. Under hypoxic conditions, cells must rapidly adapt to sustain their physiological functions. mtROS stabilize hypoxia-inducible factors (HIFs), promoting the accumulation of HIF1/2α subunits and facilitating the transcription of genes required for hypoxic adaptation (Chandel et al., 1998; Chandel et al., 2000). Additionally, mtROS activate the Protein Kinase B (PKB/AKT) Signaling Pathway by targeting Phosphatase and Tensin Homolog (PTEN), enhancing cellular survival under stress (Kim et al., 2018). mtROS also play a crucial role in cell differentiation, decisively influencing cell fate. For example, elevated mtROS levels during early differentiation stages in human cells can inhibit endodermal lineage differentiation, a process that can be fully reversed by the expression of mitochondrial-targeted peroxidases (Oka et al., 2022). However, excessive mtROS can cause oxidative stress, damaging cellular structures and functions, thereby contributing to disease progression. By oxidizing DNA bases (especially guanine), mtROS can induce replication-dependent base-pairing errors, leading to genetic mutations and increasing the risk of cancer and other diseases (Palma et al., 2024). Excessive mtROS are also closely associated with cellular aging and apoptosis (Scialo and Sanz, 2021; Zhong et al., 2017). For instance, in tumor necrosis factor-induced necroptosis, mtROS-driven Receptor-Interacting Protein Kinase 1 (RIPK1)autophosphorylation is crucial for recruiting Receptor-Interacting Protein Kinase 3 (RIPK3) to the necrosome, triggering necroptotic cell death (Zhang et al., 2017).

Internal mitochondrial changes and external environmental factors can disrupt the, ETC, leading to mtROS overproduction due to electron leakage (Palma et al., 2024). Damage or mutations in mitochondrial DNA (mtDNA) affecting genes that encode, ETC components or other mitochondrial proteins can reduce electron transport efficiency and increase ROS production. For instance, mutations in mtDNA encoding complex I subunits, which impact the CoQ10 binding pocket, are associated with Leber’s hereditary optic neuropathy. These mutations disrupt electron transfer between complex I’s Fe-S center and CoQ10, causing excessive reduction of Fe-S clusters, electron leakage, and increased production of oxygen radicals (Nunnari and Suomalainen, 2012). Furthermore, inhibiting mitochondrial fusion can result in OXPHOS defects, mtDNA loss, and mitochondrial motility impairments. Similarly, defects in mitochondrial division can also impair OXPHOS and significantly elevate ROS levels (Nunnari and Suomalainen, 2012).

External factors like immune responses and inflammation often induce mitochondrial oxidative damage. For instance, AMP-activated Protein Kinase (AMPK) and Hypoxia-Inducible Factor 1-Alpha (HIF-1α) help maintain mtROS balance in immune cells, while Mammalian Target of Rapamycin (mTOR) inhibition upregulates mtROS production and activates immune functions (Silwal et al., 2020). Impaired clearance of mtROS can lead to their accumulation. Under physiological conditions, mitochondria utilize both enzymatic and non-enzymatic defense systems to eliminate excess ROS. Enzymatic defenses include superoxide dismutase (SOD), catalase (CAT), glutathione peroxidase (GPX), and others, which convert ROS into less harmful molecules (Wang et al., 2023; Li X. et al., 2013). Manganese superoxide dismutase (MnSOD) converts superoxide anions into hydrogen peroxide, which can then participate in the Haber-Weiss or Fenton reactions to produce hydroxyl radicals (Wang et al., 2023). Metabolic disorders or dysfunctions in these systems can lead to mtROS accumulation. Excessive fatty acid oxidation (FAO) causes an overly reduced FAD pool, enhancing electron donation to the, ETC, which in turn increases mitochondrial superoxide production and mtROS levels, contributing to oxidative stress. Key enzymes like Carnitine Palmitoyltransferase 1A (CPT1A)regulate FAO and help prevent mtROS overproduction by controlling fatty acid entry into mitochondria (Wang C. et al., 2019). Additionally, metal ion homeostasis, particularly in relation to mitochondrial iron concentrations, is critical for regulating mtROS production. Metal ions such as iron and copper participate in the Fenton and Haber-Weiss reactions, catalyzing the conversion of hydrogen peroxide into highly reactive hydroxyl radicals (Paul et al., 2017; Li et al., 2021). These hydroxyl radicals can cause significant oxidative damage to cellular components, further disrupting mitochondrial redox homeostasis and contributing to mtROS accumulation (Paul et al., 2017; Li et al., 2021).

**FIGURE 2:**
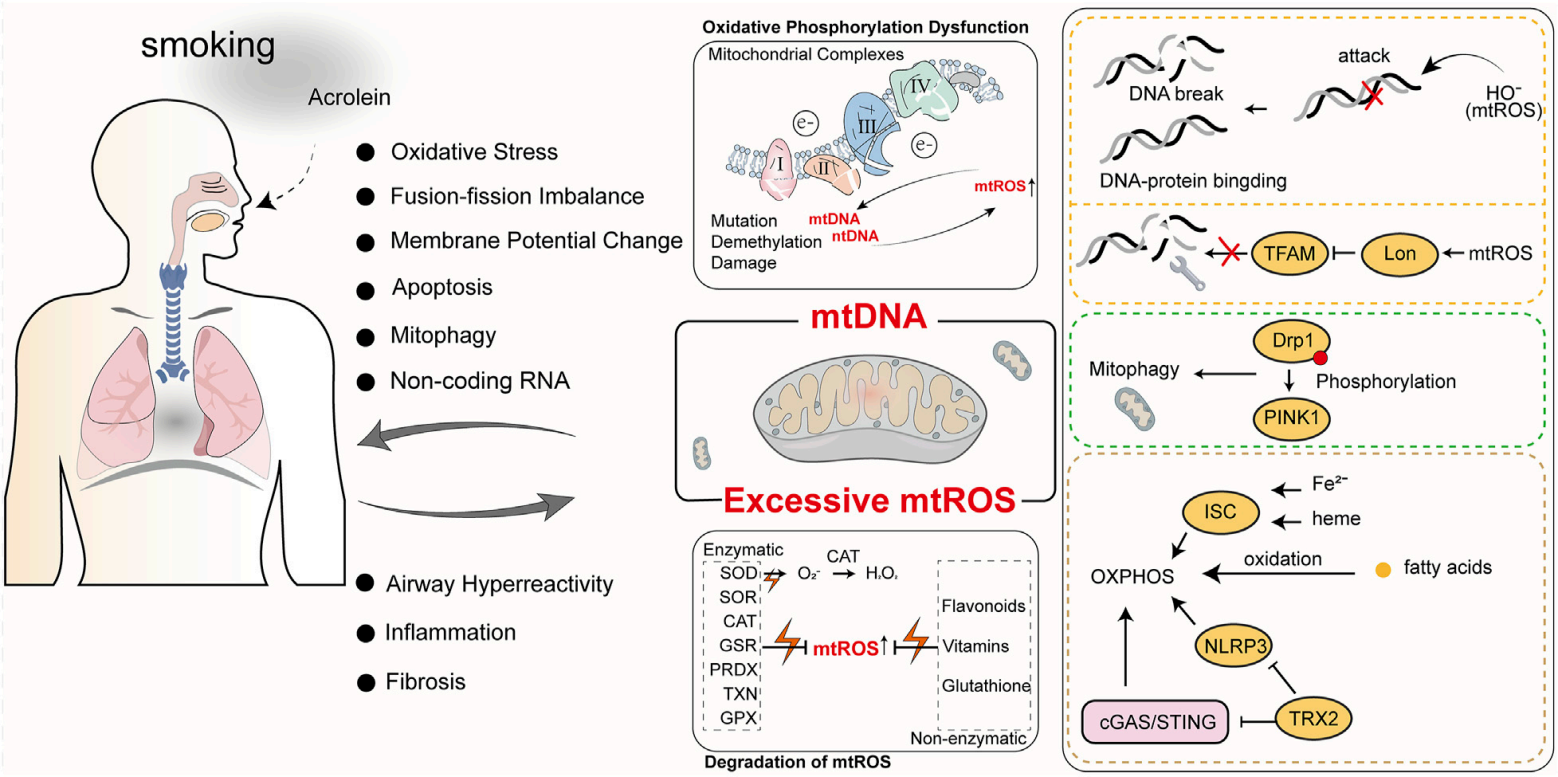
The roles of mtROS and mtDNA in COPD. Cigarette smoke is a primary cause of COPD. The harmful components of cigarette smoke, such as acrolein, can induce mitochondrial oxidative stress and mtDNA damage, ultimately impacting normal mitochondrial physiological functions. Abnormal mitochondrial function not only exacerbates COPD but also generates more mtROS and mtDNA, further impairing mitochondrial function and creating a vicious cycle.

The complex interplay of the above factors contributes to a highly intricate relationship between mtROS and the progression of COPD. While normal mtROS levels are crucial for maintaining homeostasis through mechanisms like autophagy, pathogen eradication, and inflammation resolution, excessive mtROS production can damage cellular structures and exacerbate COPD progression (Vezina and Cantin, 2018). Cigarette smoke (CS), a major cause of COPD, is closely linked to abnormal mtROS accumulation. Cigarette smoke extract (CSE) induces mtROS accumulation in pulmonary epithelial cells, promotes phosphorylation of Dynamin-Related Protein 1 (Drp1) at Serine 616(Ser616), stabilizes the mitophagy regulator PTEN-Induced Kinase 1(PINK1), and triggers mitophagy, leading to cell death and worsening COPD (Mizumura et al., 2014). In COPD mouse models, mtROS inhibition has been shown to reduce airway hyperreactivity and pulmonary inflammation, underscoring its central role in disease pathogenesis (Wiegman et al., 2015a). In COPD patients, cigarette smoke-induced mtROS cause alterations in mitochondrial fission and fusion proteins, increase oxidative stress-related gene expression, and reduce MMP and ATP levels, leading to impaired mitophagy homeostasis and mitochondrial quality control failure (Ballweg et al., 2014a; Hara et al., 2013; Fairley et al., 2023). Recent studies have shown that treating mice with Mitochondria-Targeted Tempo (MitoTEMPO), a targeted mitochondrial antioxidant, decreases mtROS levels, which in turn reduces ozone-induced pulmonary inflammation scores and attenuates inflammatory cytokine levels (Li et al., 2018). Non-coding RNAs play a crucial role in mtROS-mediated oxidative stress. miR-449b-5p suppresses lactoperoxidase (LPO) expression through miRNA-mediated post-transcriptional silencing (Santiago et al., 2024). LPO is a key component of the innate immune system in the airways, and its inhibition leads to elevated H2O2 levels, which disrupts mitochondrial homeostasis, promotes epithelial cell senescence, and enhances the production of pro-inflammatory cytokines (Santiago et al., 2024). These findings underscore the importance of regulating mtROS and related pathways, offering new targets and directions for developing future COPD treatment strategies. We have summarized the key mechanisms by which mtROS induces mitochondrial oxidative damage, ultimately contributing to the progression of COPD (Figure 2).

### 3.2 mtDNA damage in COPD

mtDNA is a distinct genetic entity within mitochondria, essential for maintaining mitochondrial function, regulating cellular metabolism, and supporting cell survival. mtDNA forms nucleoids, structures composed of proteins and evenly distributed within the mitochondrial matrix, which encode critical proteins for OXPHOS. These proteins are vital for assembling mitochondrial respiratory complexes, crucial for mitochondrial function regulation. Damage, defects, or mutations in mtDNA can lead to various diseases and are pivotal in influencing mitochondrial quality control (Yan et al., 2019).

ROS-mediated oxidative damage is the predominant form of mtDNA injury. Excessive oxidative stress can overwhelm mitochondrial repair mechanisms, leading to mtDNA mutations and subsequent mitochondrial dysfunction. This oxidative stress damages mtDNA bases, phosphodiester bonds, and deoxyribose. Research indicates that ROS, particularly hydroxyl radicals (HO-), directly assault mtDNA bases—primarily guanine and cytosine—causing strand breaks, excessive crosslinking of DNA-DNA and DNA-protein, and forming at least 20 distinct modified base adducts (Lovell and Markesbery, 2007). Moreover, mtROS, by promoting Lon protease-mediated degradation of mitochondrial transcription factor A (TFAM), substantially impairs mtDNA replication, thereby compromising mtDNA repair capabilities (Zhao et al., 2021). Removing ROS could shield mtDNA from such damage and prevent its escape. In brown adipose tissue, a deficiency in thioredoxin-2, which scavenges mtROS, mitigates excessive ROS accumulation by inhibiting the Cyclic GMP-AMP Synthase (cGAS)/Stimulator of Interferon Genes (STING) and NOD-like Receptor Pyrin Domain Containing 3(NLRP3)inflammasome pathways, thereby protecting mitochondrial membrane integrity and reducing mtDNA oxidative damage and escape (Huang et al., 2022). The Mitochondrially Encoded ATP Synthase Membrane Subunit 6 (MT-ATP6) gene, encoding the MT-ATP6 subunit in mtDNA, harbors the mutation m.9176T>C, which serves as an example of a mutation mediated by oxidative damage to mtDNA. This mutation leads to severe dysfunction in mitochondrial OXPHOS and is a primary cause of the devastating Leigh syndrome (Baertling et al., 2014). Elevated ROS levels are linked to mtDNA epigenetic control dysregulation, potentially leading to mtDNA demethylation (Sanyal et al., 2018). Although mtDNA is more prone to mutations than nuclear DNA (nDNA) due to its proximity to ROS and lack of histone protection, mitochondrial nucleoid DNA-binding proteins afford some defense, with evidence suggesting these proteins can protect mtDNA from X-ray radiation and hydrogen peroxide, akin to histones (Guliaeva et al., 2006).

mtDNA is implicated in the onset and progression of COPD. Research reveals significant correlations between plasma-free mtDNA levels and baseline characteristics of COPD patients, with notably higher levels in individuals with mild or moderate COPD compared to smokers without airflow obstruction or those with severe COPD (Zhang et al., 2021). Extended exposure to tobacco smoke and environmental pollutants significantly escalates mtDNA damage, precipitating mitochondrial dysfunction that reduces cellular energy production and heightens oxidative stress, thereby advancing COPD. Specifically, mtDNA damage diminishes mitochondrial respiratory chain efficiency, increasing mtROS production. These mtROS not only exacerbate mtDNA damage but also activate inflammatory and cell death pathways, intensifying lung damage. Studies show that exposure to high concentrations of cigarette smoke decreases MMP, augments oxidative stress, disrupts mitochondrial dynamics, and instigates the release of mtDNA into extracellular vesicles, inducing cellular aging and inflammatory response activation (Giordano et al., 2022). Furthermore, reactive unsaturated aldehydes like acrolein target mtDNA, inhibiting its replication, causing damage, and triggering apoptosis, affecting mitochondrial energy transformation and stress responses, crucial in COPD progression (Wang H. T. et al., 2017). These mitochondrial dysfunctions directly correlate with primary COPD symptoms such as chronic cough and dyspnea. Prolonged oxidative stress and inflammation alter airway and alveolar structures and promote airway smooth muscle hyperplasia and fibrosis, exacerbating COPD symptoms (Wang H. T. et al., 2017). Recent research indicates that miRNA dysregulation plays a crucial role in mitochondrial dysfunction in various lung diseases, including COPD. miR-542-3p/5p can inhibit the expression of 12S ribosomal RNA, thereby enhancing Transforming Growth Factor Beta 1 (TGF-β1) signaling, which indicates mitochondrial ribosomal stress (Garros et al., 2017). Hence, mtDNA damage and mutations are not merely indicators of COPD’s pathophysiological changes but potential targets for future therapies. Protecting mtDNA from damage, mitigating oxidative stress, and enhancing mitochondrial function could offer new treatment avenues to alleviate COPD symptoms and manage disease progression. This necessitates a thorough understanding of mtDNA damage, its repair mechanisms, and their roles in COPD, to develop targeted interventions that improve COPD patient outcomes. The role of mtDNA in oxidative damage and its contribution to COPD progression (Figure 2).

## 4 Mechanisms of mitochondrial quality control

### 4.1 Mitophagy

Under stimuli such as ROS, nutrient deficiency, and cellular aging, mitochondrial depolarization occurs in cells, leading to mitochondrial damage. In order to maintain the stability of the mitochondrial network and the stability of the intracellular environment, cells selectively engulf and degrade damaged or dysfunctional mitochondria using the autophagy mechanism. Mitophagy mainly consists of four steps: 1) Damaged mitochondria lose membrane potential, which is a prerequisite for mitophagy to occur; 2) Mitochondria are engulfed by the double-membrane vesicles of autophagosomes to form mitophagosomes, encapsulating damaged mitochondria within double-membrane vesicles; 3) Components of the mitochondria to be degraded are transported by autophagosomes to lysosomes for degradation; 4) Lysosomal or vacuolar acidic hydrolases enter autophagosomes to degrade mitochondria, and their contents are recycled (Lu et al., 2023). Mechanistically, mitophagy is mainly mediated by two pathways: ubiquitin (Ub)-dependent pathway and Ub-independent pathway.

In the Ub-dependent pathway, Parkin-dependent mitophagy is considered the major pathway of mitophagy, mainly consisting of three elements: mitochondrial damage sensor (PINK1), signal amplifier (Parkin), and signal effector (ubiquitin chain) (Harper et al., 2018). In cells with healthy mitochondria, Parkin is diffusely distributed in the cytoplasm in a self-inhibited form (Chaugule et al., 2011). Upon mitochondrial damage, PINK1 is stabilized on the outer mitochondrial membrane and activated through autophosphorylation. Activated PINK1 phosphorylates Ub to form pSer65-Ub, which recruits Parkin to the damaged mitochondria. Parkin is then fully activated by phosphorylation from PINK1, initiating a feed-forward ubiquitination process on the mitochondrial outer membrane, ultimately leading to the degradation of mitochondria through proteasomal or lysosomal pathways (Harper et al., 2018).

**FIGURE 3:**
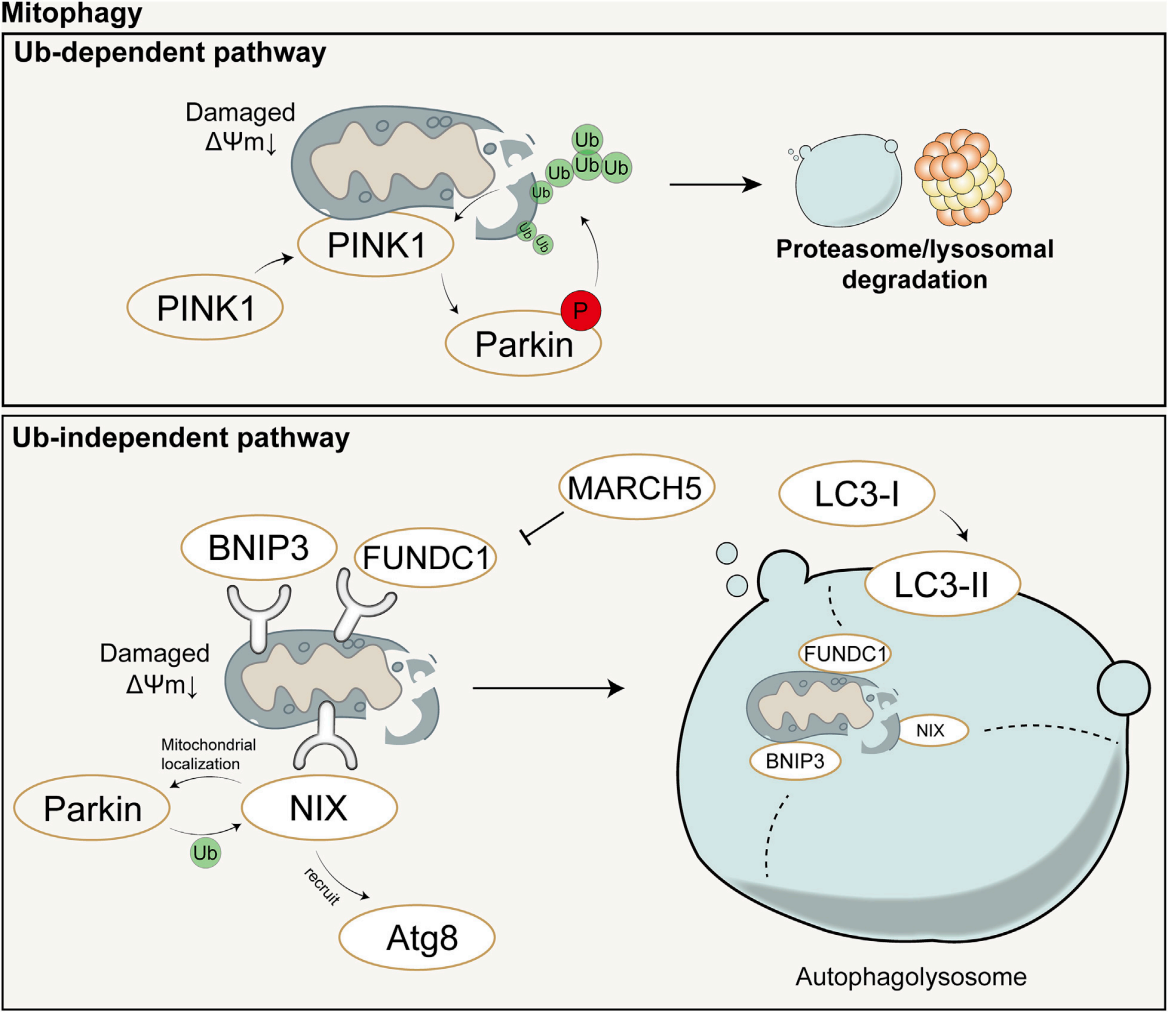
Schematic diagram of Ub-dependent mitophagy (up) and Ub-independent mitophagy (down). In the Ub-dependent pathway, Parkin-dependent mitophagy is considered the major pathway of mitochondrial autophagy, mainly consisting of three elements: mitochondrial damage sensor (PINK1), signal amplifier (Parkin), and signal effector (ubiquitin chain). In the Ub-independent pathway, mitophagy is primarily mediated by numerous autophagy receptors and ultimately completed with the aid of autolysosomes. Ub: Ubiquitin; P: Phosphorylation; ΔΨm: mitochondrial membrane potential.

The Ub-independent pathway-mediated mitophagy differs. Numerous autophagy receptors are present on the mitochondrial outer membrane, containing microtubule-associated protein 1A/1B-light chain 3 (LC3)-interacting region domains that can directly bind to LC3 without ubiquitination, thereby initiating mitophagy (Lu et al., 2023). LC3 exists in the cytoplasm in the form of LC3-I and is converted to the membrane-bound form LC3-II during autophagosome formation, accumulating on the membrane of autophagosomes and enveloping damaged mitochondria inside (!!! INVALID CITATION). The formed autophagosomes then fuse with lysosomes to form autolysosomes (!!! INVALID CITATION). During this process, the contents inside the vesicles, including damaged mitochondria, are degraded by the enzymes in lysosomes (!!! INVALID CITATION). In mammals, these receptors mainly include Nip3-like protein X (NIX)/BCL2-interacting protein 3 like (BNIP3L), BCL2-interacting protein 3 (BNIP3), FUN14 domain containing 1 (FUNDC1), etc. Both NIX and BNIP3 belong to the BCL-2 family, but they have different Bcl-2 Homology 3(BH3) domains from BCL-2 (Field and Gordon, 2022). NIX can induce mitophagy through multiple mechanisms: 1) NIX can promote the transport of Parkin to mitochondria, and Parkin can also promote the ubiquitination of NIX; 2) NIX can recruit members of the Autophagy-related protein 8(Atg8) family to damaged mitochondria and further induce mitophagy (Wei et al., 2015); 3) NIX induces mitophagy by binding to proteins associated with autophagosomes (Zhang C. et al., 2023). FUNDC1 is a mitochondrial outer membrane protein that can induce Parkin-independent mitophagy in mammalian cells under hypoxic conditions by interacting with LC3 (Liu et al., 2012). However, Membrane Associated Ring-CH-Type Finger 5 (March5), as an E3 ubiquitin ligase, can regulate hypoxia-induced mitophagy by ubiquitinating FUNDC1 for degradation (Chen Z. et al., 2017). Therefore, FUNDC1-mediated mitophagy is regulated by both ubiquitination and phosphorylation. Schematic diagram of mitophagy (Figure 3).

### 4.2 Mitochondrial dynamics

Mitochondrial dynamics refers to the continuous process of mitochondrial fusion and fission, regulated by relevant proteins, that allows for dynamic changes in mitochondrial morphology and maintains the stability of the mitochondrial network structure (Chan, 2020). Mitochondrial fusion and fission are two fundamental biological processes of mitochondria that play important roles in regulating aspects such as the quantity, shape, and function of mitochondria within cells, and they continuously fuse and divide to meet the metabolic demands of the cell. Mitofusin (Mfn) is a fusion protein with a GTPase domain located on the outer membrane of adjacent mitochondria, and its overexpression can induce mitochondrial fusion and perinuclear clustering (Santel and Fuller, 2001). Fusion of the mitochondrial outer membrane involves an irreversible GTPase-driven fusion reaction, primarily based on homotypic or heterotypic oligomeric complexes of Mfn1 and Mfn2, and proceeds through docking, tethering, and elongation steps to achieve contact with the mitochondrial outer membrane (Ishihara et al., 2004; Eura et al., 2003). Lack of Mfn1 or Mfn2 leads to embryonic lethality in mouse models or severe genetic diseases in humans (Chen et al., 2003; Giacomello et al., 2020). Fusion of the mitochondrial inner membrane differs from that of the outer membrane. Optic Atrophy 1(Opa1) is the only dynamin-like protein identified in the mitochondrial inner membrane to date, which is crucial for mitochondrial elongation and enables mitochondria about to fuse to share cellular contents (Song et al., 2009). Only one of the mitochondrial inner membranes of the two mitochondria to be fused requires Opa1 to drive fusion (Song et al., 2009). Opa1 possesses an N-terminal domain which includes a mitochondrial targeting sequence for mitochondrial import, a transmembrane domain anchoring it to the inner mitochondrial membrane, and a helical coiled-coil domain (Olichon et al., 2002). Lack of Opa1 results in mitochondrial fragmentation, reduced cristae, and OXPHOS, and similarly leads to embryonic lethality in mouse models (Cai et al., 2022; Quintana-Cabrera and Scorrano, 2023). The majority of Opa1 protein is exposed to the intermembrane space, including the GTPase domain, middle domain, and GTPase effector or assembly domain (Olichon et al., 2002). Opa1 can undergo selective splicing, generating eight different transcripts in humans, all of which are anchored to the inner mitochondrial membrane (Ishihara et al., 2006). Complementary and shared transcripts can even rescue mitochondrial dysfunction caused by mtDNA mutations (Quintana-Cabrera and Scorrano, 2023).

**FIGURE 4:**
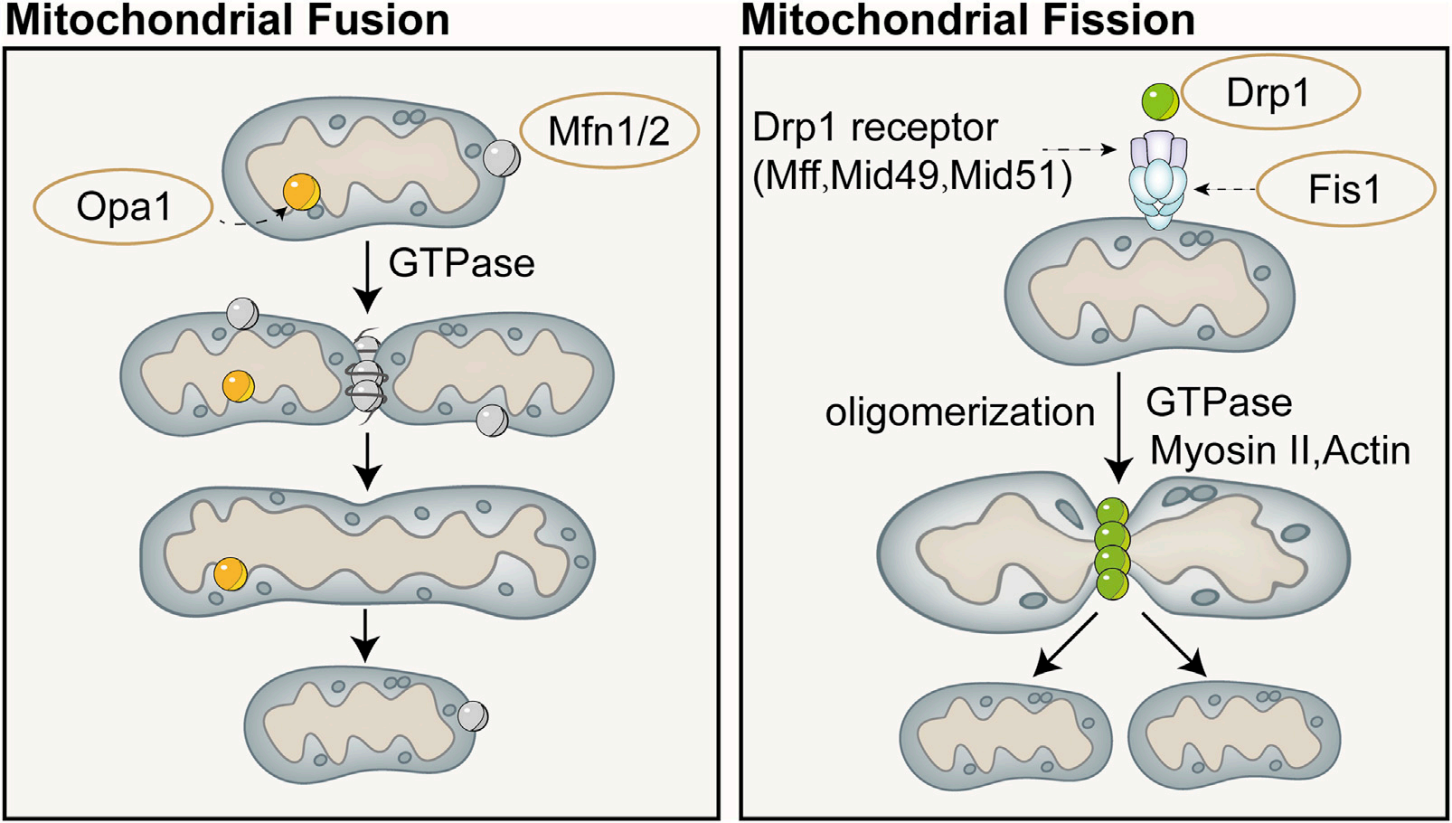
Schematic diagram of mitochondrial fusion (left) and fission (right). Mitochondrial fusion is categorized into fusion of the outer mitochondrial membrane and the inner mitochondrial membrane. Fusion of the outer mitochondrial membrane primarily involves an irreversible fusion reaction mediated by Mfn and driven by GTPase. Opa1 is the only dynamin-like protein identified in the mitochondrial inner membrane to date, which is crucial for mitochondrial elongation and enables mitochondria about to fuse to share cellular contents. Mitochondrial division is primarily mediated by the GTPase dynamin-related protein Drp1, which can both constrict tubular membranes and is the minimal component sufficient for membrane separation.

Mitochondrial division is primarily mediated by the GTPase Drp1, which can both constrict tubular membranes and is the minimal component sufficient for membrane separation (Kamerkar et al., 2018). The endoplasmic reticulum wraps around mitochondria and contracts in regions of approximately 300–500 nm in diameter, which will accommodate approximately 120 nm in diameter of Drp1 (van der Bliek et al., 2013). On the surface of the endoplasmic reticulum, actin interacts mainly with inverted formin 2 (INF2) located on the endoplasmic reticulum. Spire-type Actin Nucleation Factor 1C (Spire1C) binds INF2 and promotes actin assembly on mitochondrial surfaces and isrupting either Spire1C actin- or formin-binding activities reduces mitochondrial constriction and division (Manor et al., 2015). In cases where the mitochondrial outer membrane has not ruptured, actin cables may provide mechanical force to drive the initial contraction of the mitochondria (Moore et al., 2016). Additionally, actin II and actin can also recruit Drp1 to the vicinity of mitochondria and induce its oligomerization (this process is mediated by mitochondrial outer membrane proteins such as mitochondrial fission factor, mitochondrial fission 1 protein, mitochondrial dynamics protein 49 (MID49), and mitochondrial elongation factor 1 (MIEF1), thereby promoting division (Quintana-Cabrera and Scorrano, 2023; Jin et al., 2021; Wang J. et al., 2020). Division will not occur or can be reversed when actin has not polymerized, whereas polymerization of actin during division plays a crucial role in exerting membrane tension (Mahecic et al., 2021). Schematic diagram of mitochondrial fusion and fission (Figure 4).

### 4.3 Mitochondrial biogenesis

Mitochondrial biogenesis is a complex biological process involving fine regulation of mitochondrial quantity and quality, including proliferation, protein synthesis, DNA replication, and remodeling of its network. This process is crucial for maintaining cellular homeostasis by controlling organelle self-renewal and mtDNA maintenance. Mitochondrial biogenesis not only ensures the health and function of mitochondria themselves but also plays a key role in meeting the cell’s energy supply, signal transduction, death regulation, and the demands of differentiation and proliferation. Cells can adapt to different metabolic demands and environmental pressures by adjusting mitochondrial biogenesis, thus optimizing energy production efficiency and maintaining the physiological function of the entire cell. Peroxisome Proliferator-Activated Receptor Gamma Coactivator 1-Alpha (PGC-1α) is considered the major regulatory factor of mitochondrial biogenesis. This pathway begins with the activation of PGC-1α in the cytoplasm, which subsequently stimulates the expression of a series of nuclear transcription factors, including nuclear respiratory factor-1, nuclear respiratory factor-2, and estrogen-related receptor-α, among others (Cameron et al., 2016). Activation of PGC-1α is mainly achieved through two pathways: one is deacetylation mediated by NAD-dependent protein deacetylase Sirtuin-1 (SIRT1). SIRT1 utilizes NAD as a coenzyme to catalyze the removal of acetyl groups from PGC-1α, promoting its nuclear translocation (Tang, 2016; Rodgers et al., 2005); The other is phosphorylation mediated by AMPK and other kinases. AMPK is activated when the cellular AMP/ATP ratio changes, capable of phosphorylating PGC-1α and promoting its transcription (Jager et al., 2007; Canto and Auwerx, 2009). Additionally, methylation mediated by protein arginine methyltransferase 1 (PRMT1) and phosphorylation by p38 and protein kinase A (PKA) can activate PGC-1α and mediate the transcription and translation of proteins related to mitochondrial biogenesis (Teyssier et al., 2005; Puigserver et al., 2001; Chang et al., 2010).

**FIGURE 5:**
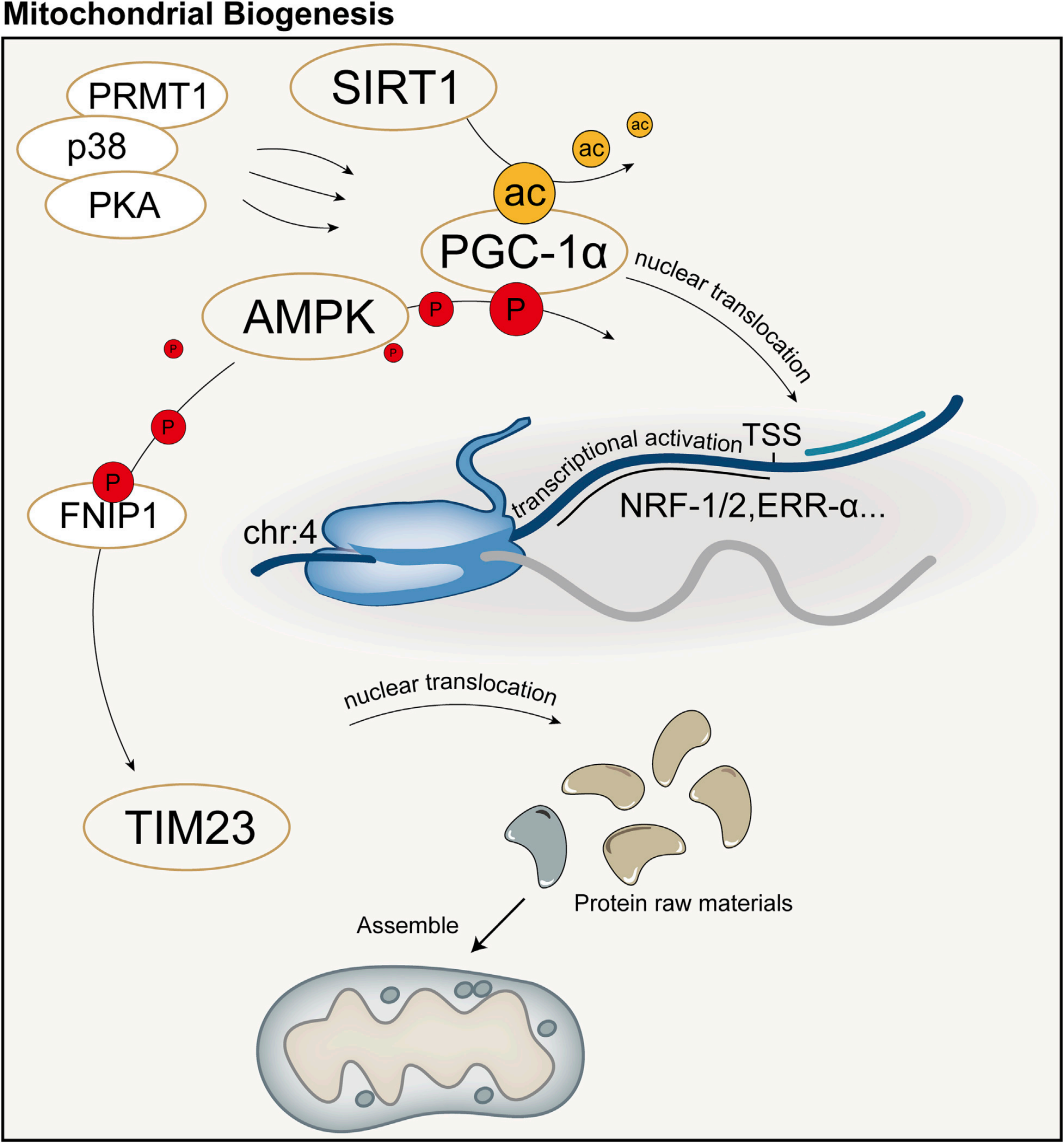
Schematic diagram of mitochondrial biogenesis. PGC-1α is considered the major regulatory factor of mitochondrial biogenesis. In the mitochondrial biogenesis process predominantly mediated by PGC-1α, the synthesis of mitochondria primarily involves transcription and translation by the nucleus, followed by subsequent synthesis and assembly. ac: acetylation; P: Phosphorylation; TSS: Transcription Start Site.

Protein precursors synthesized in the cytoplasm can be guided to the mitochondrial matrix by the translocase of the inner membrane 23 (TIM23), assembled inside mitochondria, and sorted to precise locations, namely the mitochondrial matrix or inner membrane, with the energy required for this process provided by the MMP and ATP (Moulin et al., 2019; Demishtein-Zohary and Azem, 2017). TFAM is a key factor for mtDNA transcription and replication, and the activation of PGC-1α can increase the expression of TFAM, directing the biogenesis program ultimately to mitochondria (Popov, 2020). Subsequently, with the assistance of specific translation factors encoded by nDNA (such as initiation factors 2 and 3, elongation factors Tu, Ts, and G1, translation release factor 1, and recycling factor), mtDNA initiates the transcription and translation process, producing the proteins required by mitochondria (Popov, 2020). Recent studies have shown that AMPK in cells can directly phosphorylate five conserved serine residues in folliculin interacting protein 1 (FNIP1), inhibiting the function of the folliculin (FLCN)-FNIP1 complex to promote transcription factor EB (TFEB) nuclear translocation, thereby inducing mitochondrial biogenesis (Malik et al., 2023). Schematic diagram of mitochondrial biogenesis (Figure 5).

## 5 Mitochondrial quality control and COPD

Mitochondrial quality control is a finely tuned process that encompasses multiple aspects such as mitophagy, mitochondrial dynamics, and mitochondrial biogenesis, with the overarching goal of maintaining the quantity, morphology, and function of mitochondria. However, in COPD, these regulatory mechanisms may be compromised due to the persistent action of inflammatory mediators or exposure to environmental stressors, leading to a failure in the timely clearance of damaged mitochondria, which subsequently exacerbates the progression of COPD (Table 1).

**TABLE 1:** Mitochondrial quality control and COPD.

Mitochondrial quality control	Research subjects	Mechanisms	Ref.
Mitophagy	CS-induced COPD mice CSE-induced BEAS-2B cells	increased PKM2; exacerbated airway inflammation and epithelial-mesenchymal transition	Li et al. (2022a)
	CS or PM2.5-induced COPD rats CS or PM2.5-induced A549 cells	increased NEAT1, PINK1, and Parkin; resulted in mitochondrial swelling; induced mitophagy; triggered the formation of autophagosomes	Lin et al. (2022)
	PM2.5-CSE induced BEAS-2B cells PM2.5-CS induced COPD mice	increased Parkin and PINK1 expression; increased mito-ROS production; enhanced mitophagy; induced NOX4/Nrf2-mediated redox imbalance	Fan et al. (2023)
	CS-induced COPD mice with *PRKN* gene-knockout	exhibited a more substantial accumulation of damaged mitochondria, cellular senescence, emphysema lesions, and inflammatory response	Araya et al. (2019)
	CS-induced COPD mice CSE-induced HFL1 cells	increased PINK1 expression; reduced Parkin translocation to damaged mitochondria; reduced ΔΨm; resulted in insufficient mitophagy and ineffective clearance of abnormal mitochondria	Ahmad et al. (2015)
	CS-induced COPD mice CSE-induced HBE cells	increased FUNDC1; activated excessive mitophagy	Wen et al. (2019)
	CSE-induced BEAS-2B cells	increased FUNDC1; reduced mitochondrial membrane potential; inhibited cell survival and promoted apoptosis	You et al. (2023)
Mitochondrial dynamics	BRPM2.5-induced 16HBE cells BRPM2.5-induced COPD mice	promoted excessive mitochondrial fission; upregulated pDRP1 protein; increased mtROS production; reduced MMP and ATP levels; increased swollen mitochondria	Gao et al. (2022)
	ATII cells isolated from patients with emphysema	reduced p-DRP1, FIS1, and MFN2 levels; exhibited severe mtDNA damage and excessive mitochondrial superoxide production	Kosmider et al. (2019)
	Primary ATII cells from patients with COPD CSE-induced A549 cells	reduced mitochondrial area and perimeter, and impaired mitochondrial cristae arrangement; reduced MFN2 and OPA1; increased mitochondrial fragmentation	Li et al. (2023)
Mitochondrial biogenesis	CS + LPS-induced COPD rats	increased ROS and PGC-1α levels	Li et al. (2010)
	Patients with COPD	reduced PGC-1α mRNA expression; reduced TFAM protein levels	Remels et al. (2007)
	CS-induced COPD mice	reduced PGC-1α; weakened grip strength; reduced hindlimb muscle mass	Zhang et al. (2022a)
	CS + *Klebsiella pneumonia* suspension-induced COPD rats	reduced AMPK-α, PGC-α, and TFAM mRNA levels; reduced mitochondrial biogenesis; led to muscle fiber atrophy	Mao et al. (2020)

### 5.1 Mitophagy and COPD

Exposure to CS is a major risk factor for COPD, and long-term exposure to CS damages bronchial epithelial cells via multiple mechanisms. According to reports, mitochondrial structure and function undergo drastic changes in BEAS-2B under continuous stimulation by CSE (Hoffmann et al., 2013). Damaged mitochondria are eliminated through the activation of mitophagy, a process that helps maintain cellular homeostasis by removing dysfunctional mitochondria. Notably, both overactivation and underactivation of mitophagy are related to the pathogenesis of COPD. As a key regulatory pathway for mitophagy, the PINK1/Parkin pathway is involved in COPD pathogenesis; However,its underlying mechanism remains elucidated. Upregulation of this pathway can lead to overactivation of mitophagy, affecting disease progress through multiple mechanisms, including disrupting energy metabolic balance, inducing programmed cell death, and exacerbating oxidative stress and inflammation through excessive mtROS production (Mizumura et al., 2014; Ko et al., 2021). Given the crucial role of mitophagy in maintaining cellular homeostasis and disease development, there is strong interest in its regulatory targets. In an experimental model of CS-induced COPD, Li et al. observed increased expression levels of pyruvate kinase M2(PKM2) in mouse lung tissue and BEAS-2B cells, which was accompanied by exacerbated airway inflammation and epithelial-mesenchymal transition; however, when PKM2 was knocked down, PINK1/Parkin-mediated mitophagy was inhibited, and the aforementioned pathological changes were reversed, suggesting that PKM2 plays a role in regulating PINK1/Parkin-mediated mitophagy (Li D. et al., 2022). Nuclear Enriched Abundant Transcript 1(NEAT1) promotes the ubiquitination and degradation of PINK1 and impairs PINK1-dependent mitophagy (Huang et al., 2020). Lin et al. discovered a regulatory mechanism between NEAT1 and mitophagy mediated by PINK1, and the increase of NEAT1, PINK1, and Parkin in the lung tissue of rats with COPD induced by CS or PM2.5,resulted in mitochondrial swelling, subsequently inducing mitophagy, and ultimately triggering the formation of autophagosomes. Moreover, these rats exhibited inflammatory changes, airway remodelling, and lung bulla formation (Lin et al., 2022). Knocked down NEAT1 decreased PINK1 and Parkin expression in A549 cells exposed to CSE and PM2.5, but increased MMP, suggesting that NEAT1 enhances mitophagy during COPD progression by upregulating PINK1 (Lin et al., 2022). A study by Fan showed that in a COPD cell model, high-dose PM2.5 was more likely to trigger oxidative stress and mitophagy in BEAS-2B cells compared with low doses. Notably, under the combined effect of PM2.5-CSE, Parkin and PINK1expression substantially increased,and the application of NADPH Oxidase 4(NOX4) siRNA effectively modulated NOX4/Nuclear Factor Erythroid 2-Related Factor 2 (Nrf2)-mediated redox reactions, suppressed ROS generation, and alleviated excessive mitophagy (Fan et al., 2023). Mice exposed to PM2.5-CS exhibited more substantial airway inflammation and mucus hypersecretion compared with those of CS only mice. Additionally, NOX4/Nrf2-mediated redox imbalance and cell apoptosis were considerably exacerbated; however, after treatment with the mitochondrial-targeted antioxidant, MitoTEMPO, the NOX4/Nrf2-mediated redox imbalance was notably improved, with suppressed ROS production and downregulated expression of PINK1 and Parkin (Fan et al., 2023).

When mitophagy is insufficient, dysfunctional mitochondria accumulate within cells, causing cellular damage through multiple mechanisms and triggering a series of diseases and pathological processes (Tsubouchi et al., 2018; Jin and Youle, 2012; Kawajiri et al., 2010). Defects or insufficiencies in PINK1/Parkin-mediated mitophagy are important factors in the progression of COPD. Parkin plays a pivotal role in mitophagy initiation., In response to mitochondrial dysfunction, PINK1 uniquely stabilizes itself on the membrane of damaged mitochondria, maintaining its kinase activity while recruiting Parkin to eliminate damaged mitochondria. However, defects or insufficiencies in Parkin can disrupt mitophagy, impeding the efficient removal of damaged mitochondria and ultimately compromising cellular function (Ashrafi and Schwarz, 2013; Youle and Narendra, 2011; Bingol et al., 2014; Vincow et al., 2013). Araya et al. discovered that, compared with wild-type COPD mice, parkin RBR E3 ubiquitin protein ligase (*PRKN*) gene-knockout mice exhibited a more substantial accumulation of damaged mitochondria, cellular senescence, airway wall thickening, emphysema lesions, and inflammatory response after exposure to CS for 6 months (Araya et al., 2019). *In vitro* experiments showed that even with reduced PINK1 protein levels, overexpression of PRKN was still capable of inducing mitophagy, effectively reducing mtROS production, and delaying cell senescence. However, PINK1 overexpression did not enhance CSE-induced mitophagy or restore the mitophagy impairment caused by PRKN knockdown, suggesting that PRKN protein levels may be the primary rate-limiting factor in PINK1/PRKN-mediated mitophagy (Araya et al., 2019). Another study found that after 6 months of CS exposure, COPD mice showed increased PINK1 expression in the lung tissue but reduced Parkin translocation to damaged mitochondria, leading to insufficient mitophagy and ineffective clearance of abnormal mitochondria (Ahmad et al., 2015). CSE treatment of Human Fetal Lung Fibroblast 1 (HFL1) cells reduced ΔΨm, severely impaired Parkin translocation, and damaged the mitophagy process, ultimately accelerating cell senescence; however, overexpression of Parkin restored mitophagy, reduced perinuclear mitochondrial accumulation, and delayed cell senescence. Notably, the positive effect of Parkin overexpression in delaying cell senescence depended on pretreatment with MitoTEMPO (Ahmad et al., 2015). These findings indicate that in the COPD model, Parkin translocation to mitochondria is impaired, leading to insufficient mitophagy (Ahmad et al., 2015). Elevated PINK1 expression may be related to the accumulation of damaged mitochondria due to reduced Parkin, which further accelerates cell senescence and exacerbates COPD progression (Ito et al., 2015).

Although the PINK1/Parkin pathway is generally regarded as the dominant mechanism regulating mitophagy, mitophagy can still be maintained in the absence of PINK1 or Parkin. FUNDC1, a novel receptor protein, has been identified as a positive regulator that promotes mitophagy (Chen Z. et al., 2017; Lampert et al., 2019). COPD pathogenesis is linked to mitophagy mediated by FUNDC1 (Wen et al., 2019; You et al., 2023). FUNDC1 is overexpressed in CS-induced COPD mice, and silencing of FUNDC1 improves lung function in mice (Wen et al., 2019). Silencing FUNDC1 in CSE-treated Human Bronchial Epithelial Cells (HBECs) inhibited the overactivation of mitophagy, thereby enhancing mitochondrial transmembrane potential and reducing Interleukin-6 (IL-6) and Tumor Necrosis Factor-Alpha (TNF-α) levels. Notably, this treatment also improved mucosal ciliary clearance, which may be substantial for COPD treatment (Wen et al., 2019). You et al. also confirmed that the expression of FUNDC1 was considerably upregulated in CSE-treated BEAS-2B cells However, when Ubiquitin-Specific Peptidase 19(USP19) was knocked down, FUNDC1 degradation increased, thus inhibiting the overactivation of mitophagy. Consequently, MMP improved, the cell survival rate increased, and cell apoptosis was effectively suppressed (You et al., 2023). Therefore, USP19 participates in mitophagy by regulating FUNDC1 (You et al., 2023). However, the FUNDC1 protein level in the vastus lateralis of patients with COPD was lower than that in healthy controls, which may be associated with increased mitochondrial breakdown in the skeletal muscles of patients with COPD, thereby reducing the number of mitochondria (Leermakers et al., 2018). These studies provide important evidence for the role of mitophagy in COPD pathogenesis. However, whether mitophagy plays a protective or detrimental role in COPD remains controversial.

### 5.2 Mitochondrial dynamics and COPD

Mitochondrial function largely depends on the balance between mitochondrial fusion and fission; once this balance is disrupted, the morphological structure of the mitochondria is impaired, leading to dysfunction (Chiu et al., 2021). Long-term CS exposure disrupted the balance between mitochondrial fission and fusion in a COPD model (Hoffmann et al., 2013). CSE-treated lung epithelial cells exhibited increased perinuclear mitochondrial clustering, shortened mitochondrial fragments, elevated Drp1, and decreased Mfn2 (Sundar et al., 2019). Similarly, CSE-treated A549 cells in a COPD cell model showed a substantial increase in Drp1 and a decrease in Mfn2 (Guan et al., 2021). Evidence suggests that excessive mitochondrial fission in dysfunctional mitochondria leads to an excessive release of ROS and cytochrome c, triggering cellular dysfunction (Chen et al., 2005; Shenouda et al., 2011). It also generates numerous functionally weakened mitochondrial fragments, increasing ROS production and decreased overall antioxidant capacity (Mishra and Chan, 2014; Knott et al., 2008). However, the accumulation of ROS exerts various negative effects on mitochondria, creating a vicious cycle (Aravamudan et al., 2014). Hara et al. revealed that CSE exposure in HBECs upregulated Drp1 and Fission 1(Fis1) expression in mitochondria; the translocation of Drp1 and Fis1 to mitochondria promoted mitochondrial fragmentation, leading to accumulation of divided mitochondria, increased ROS production, and accelerated cellular senescence (Hara et al., 2013). Notably, BRPM2.5 also impaired mitochondrial dynamics. Gao et al. reported that in 16HBE cells, BRPM2.5 treatment promoted excessive mitochondrial fission by upregulating phosphorylated dynamin-related protein 1 (p-Drp1) protein expression, accompanied by mitochondrial dysfunction, such as increased mtROS production, decreased MMP, and reduced ATP levels, which exacerbated inflammation and increased cell death (Gao et al., 2022). In the lung tissue of COPD mice induced by BRPM2.5, the number of swollen mitochondria increased, and mitochondrial cristae were depleted (Gao et al., 2022). Skeletal muscle dysfunction is a key pathophysiological feature of patients with COPD. Tan et al. found that in CSE-treated quadriceps femoris cells, Drp1 expression substantially increased, accompanied by increased ROS release and apoptosis. Drp1 expression increased with higher CSE concentrations, suggesting that CSE may promote excessive mitochondrial fission by upregulating Drp1, thus affecting cellular energy metabolism (Tan et al., 2021). However, moderate mitochondrial fission maintains mitochondrial function and cellular homeostasis by activating mitophagy to selectively eliminate damaged mitochondrial components (Ni et al., 2015). Alveolar Type II Cells (ATII) isolated from patients with emphysema exhibited severe mtDNA damage and excessive mitochondrial superoxide production, along with reduced p-Drp1, FIS1, and MFN2 levels, suggesting that mtDNA damage leads to abnormal mitochondrial dynamics (Kosmider et al., 2019). Patients with emphysema showed reduced mitochondrial fission compared with that of smokers and non-smokers, potentially impeding mitophagy, leading to mitochondrial dysfunction and ATII cell death (Kosmider et al., 2019). Drp1 expression in COPD models exhibits conflicting findings, possibly owing to the varying severity of COPD, different exposure factors, and inconsistent exposure durations. Overall, mitochondrial fragmentation caused by fission. is crucial in COPD pathogenesis.

Mitochondrial fusion is considered the self-protection mode of damaged organelles, which helps maintain the normal function of mitochondria by promoting the production of networked and elongated mitochondria and plays a vital role in maintaining the stability of mtDNA and improving the efficiency of ATP synthesis, thus helping to restore energy supply in cells (Chen et al., 2010). Impaired mitochondrial fusion can increase mitochondrial fragmentation, adversely affecting mitochondrial function (Ni et al., 2015). In a model of airway epithelial cell injury induced by CSE, the expression of mitochondrial fusion proteins (MFN2 and OPA1) was reduced, accompanied by enhanced oxidative stress and inflammatory response (Wang M. et al., 2019). Compared with non-smokers, primary ATII cells from patients with COPD exhibited diverse structural changes, such as reduced mitochondrial area and perimeter, and impaired mitochondrial cristae arrangement. These changes not only resulted in a substantial decrease in mitochondrial activity, but were also coupled with a reduction in the expression of MFN2 and OPA1, exacerbating impaired mitochondrial fusion and increasing mitochondrial fragmentation (Li et al., 2023). Promoting mitochondrial fusion by upregulating MFN2 and OPA1 in CSE-treated A549 cells effectively reduced mtROS production, inhibited excessive mitophagy, and delayed cellular aging (Li et al., 2023). COPD is commonly associated with aging, and the disruption of mitochondrial dynamics promotes cellular aging. In HBECs, the increase in ROS levels triggered by CSE exposure was likely a key factor leading to the translocation of Drp1 to the mitochondria and subsequent induction of mitochondrial fission, and effective inhibition of Drp1 translocation and mitochondrial fragmentation by the ROS scavenger N-acetylcysteine (NAC) supported this view (Hara et al., 2013). Drp1 knockdown also substantially suppressed CSE-induced mitochondrial fragmentation; however, knockdown of OPA1 and MFN fusion proteins induced mitochondrial fragmentation, increased mtROS production, and thereby accelerated HBECs aging. Combined treatment with NAC and MitoTEMPO substantially inhibited cellular aging (Hara et al., 2013). Notably, a study has brought us a brand-new discovery (Ballweg et al., 2014b). When mouse lung epithelial 12 (MLE12) cells were treated with non-toxic doses of CSE, considerably elongated, with a transient increase in Mfn2, indicating the occurrence of mitochondrial fusion. These results imply that enhancing mitochondrial fusion may be an adaptive response of cells to low-dose CSE treatment (Ballweg et al., 2014b). This further confirmed, to a certain extent, that the response of cells to cigarettes was indeed closely related to the dose. In summary, strategies to reduce mitochondrial fission and promote fusion may be effective in maintaining mitochondrial homeostasis and function and restoring cellular energy supply, and may represent a promising therapeutic approach for delaying COPD progression.

### 5.3 Mitochondrial biogenesis and COPD

Mitochondrial biogenesis is crucial for maintaining cellular homeostasis and it enables cells to replace damaged or aged mitochondria with new ones, thus maintaining normal morphology, structure, and function of mitochondria. PGC-1α, a key regulatory factor for mitochondrial biogenesis, is central in regulating mitochondrial function and cellular energy metabolism (Halling and Pilegaard, 2020). PGC1-α levels gradually decreased in lung tissues of patients with moderate and severe COPD compared with that of healthy controls (Li et al., 2010). This change was presumably associated with intensified inflammatory response, oxidative stress, and hypoxia, leading to severe mitochondrial dysfunction beyond mitochondrial quality control capabilities, directly impacting the expression and activity of PGC1-α (Li et al., 2010). Notably, PGC-1α levels were elevated in the lung tissues of patients with mild COPD. These patients initiated compensatory mechanisms in the early stages of the disease to maintain mitochondrial function and energy supply, coping with inflammatory damage and oxidative stress (Li et al., 2010). This compensatory mechanism may help alleviate COPD symptoms and delay disease progression. Oxidative stress plays a pivotal role in the pathogenesis of COPD. Excessive ROS damage cells and tissues under oxidative stress and accelerate disease progression (Kume et al., 2023). Mitochondrial biogenesis induced by PGC1-α can partially restore mitochondrial function of cells temporarily exposed to oxidative stress. In a CS + lipopolysaccharide-induced COPD rat model, Li et al. observed substantially higher ROS and PGC-1α levels in lung tissues compared to those in the blank control group. The levels of ROS and PGC-1α showed a positive correlation, suggesting that the upregulation of PGC1-α may be an adaptive response to oxidative stress (Kume et al., 2023). Skeletal muscle dysfunction is a common complication in patients with COPD, manifesting as muscle fiber reduction and proportional imbalance of various types of fibres (Barreiro and Sznajder, 2013; Lewis et al., 2012). These pathological changes lead to decreased muscle strength and tolerance, resulting in reduced exercise tolerance and a decline in the patient’s quality of life (Rutten et al., 2006; Gosselink et al., 1996). PGC-1α dysregulation is involved in COPD-related skeletal muscle dysfunction (Remels et al., 2007). Compared with healthy controls, patients with COPD had lower PGC-1α mRNA expression in skeletal muscle, accompanied by reduced TFAM protein levels, which may be linked to decreased muscle oxidation capacity in COPD (Remels et al., 2007). Similarly, Zhang et al. observed that after 24 weeks of CS exposure, the expression of PGC-1α in the skeletal muscle of COPD mice decreased, accompanied by weakened grip strength and a substantial reduction in hindlimb muscle mass in the mice (Zhang L. et al., 2022). Pan also observed that the content of PGC-1α in the gastrocnemius of COPD model rats was closely related to the duration of exposure to chronic intermittent hypoxia-hypercapnia (CIHH) (Pan et al., 2016). Compared with the blank control group, the expression of PGC-1α in the gastrocnemius of rats substantially increased after 2 weeks of CIHH exposure; however, with prolonged exposure time, the expression of PGC-1α decreased after 4 weeks, accompanied by muscle fiber shift and injury, leading to a decrease in running ability of rats. The decline of PGC-1α was effectively reversed in rats after electrical stimulation of gastrocnemius (Pan et al., 2016). The disease course affects PGC-1α levels, consistent with Li’s findings (Li et al., 2010). AMPK is an important regulatory factor in cellular energy metabolism and is crucial for regulating mitochondrial biogenesis (Zhang et al., 2018). AMPK activation has great potential for improving skeletal muscle dysfunction (Ozaki et al., 2023; Li Q. et al., 2022). AMPK-α, PGC-α, and TFAM mRNA levels in the skeletal muscle of COPD rats substantially decreased, resulting in the reduction of mitochondrial biogenesis and muscle fiber atrophy. Nevertheless, the Bufei Jianpi formula effectively promoted mitochondrial biogenesis by upregulating the expression of AMPK, thereby substantially improving skeletal muscle dysfunction in COPD rats (Mao et al., 2020).

## 6 Therapeutic strategy

Given the vital role of mitochondrial dysfunction in COPD pathogenesis, interventions targeting mitochondrial quality control mechanisms to preserve and restore mitochondrial function have emerged as crucial therapeutic strategies to prevent and treat COPD. Various compounds targeting mitochondrial quality control mechanisms have been proven effective in halting the progression of COPD, significantly advancing the drug development process for COPD patients and demonstrating promising therapeutic prospects (Table 2).

**TABLE 2:** Various compounds targeting mitochondrial quality control mechanisms to treat COPD.

Therapeutic strategy	Compounds	Dosage	Research subjects	Effects/mechanisms	Ref.
Targeting mtROS	MitoQ	100 nmol/L	CSE-induced HUVECs	suppressed ROS production and excessive autophagy; reduced mitochondrial damage	Chen et al. (2019)
	MitoQ	5 mg/kg (*invivo*) Not mentioned (*in vitro*)	Ozone-induced COPD mice H2O2-exposed ASM cells	decreased mtROS levels; increased ATP; inhibited the proliferation of ASM cells	Wiegman et al. (2015b)
	MitoTEMPO	100 nM to 1 μM	CSE-induced HFL1 cells; CSE-induced SAECs	reduced mtROS; decreased perinuclear accumulation of damaged mitochondria; inhibited cellular DNA damage; partially delayed cellular senescence	Ahmad et al. (2015)
	MitoTEMPO	100 μM	CSE-induced HBECs	inhibited mitochondrial fragmentation by reducing mtROS levels; delayed HBECs senescence	Hara et al. (2013)
	SS-31	5 μM	BRPM2.5-induced 16HBECs	lowered mtROS levels; promoted ATP production; suppressed inflammatory response	Gao et al. (2022)
Targeting mitophagy/autophagy	Melatonin	2.5,5,10,20 mg/kg (*in vivo*) 40 μM(*in vitro*)	CS-induced COPD mice CSE-induced L-132 cells	stimulated mitophagy to increase the clearance of damaged mitochondria; inhibited ER stress; inhibited oxidative stress	Mahalanobish et al. (2020)
	SRT1720	4 mM	CSE-induced BEAS-2B cells	enhanced mitophagy by decreasing FOXO3 acetylation and increasing PINK1; reduced cell apoptosis and oxidative stress	Jiang et al. (2022)
	Pirfenidone	50 μg/mL	CSE-induced HBECs	induced PRKN protein expression, thereby enhancing mitophagy; prevented the accumulation of mtROS and cell senescence	Araya et al. (2019)
	Quercetin	5,10,20 μM	CSE-induced BEAS-2B cells	increased p-DRP-1 and PINK1 expression; increased functional mitochondria and reduced mtROS production	Son et al. (2018)
	Thymoquinone	20,50 μM	CSE-induced BEAS-2B cells	increased PINK1 and p-Drp levels improved cell viability	Dera et al. (2020)
	Puerarin	50,100,200 μg/mL	CSE-induced HBECs	decreased PINK1 and Parkin expression; improve mitochondrial damage; increase ATP; reduced ROS level	Wang et al. (2022a)
	3-MA	4 mg/mL (*in vivo*) 4 mM(*in vitro*)	CS-induced COPD mice CSE-induced THP-1cells	inhibited autophagy; ameliorated airway inflammation	Huang et al. (2021)
	3-MA	20 mg/kg	CS-induced COPD mice	decreased LC3B level; inhibited formation of autophagosome	Liu et al. (2021)
Targeting mitochondrial dynamics	Mdivi-1	50 mg/kg (*in vivo*) 50 μM*(in vitro)*	CS-induced COPD mice CSE-induced Beas-2B cells	inhibited mitochondrial fission; inhibited mitophagy; reversed the decrease of ΔΨm	Mizumura et al. (2014)
	Sodium tanshinone IIA sulfonate	1,2.5.5 μM	CSE-induced A549 cells	decreased Drp1 protein; increased Mfn2 protein; prevented the loss of Δψm; inhibited apoptosis	Guan et al. (2021)
Targeting mitochondrial biogenesis	GHK-Cu	0.2.2 mg/kg (*in vivo*) 5.10 μM *(in vitro)*	CS-induced COPD mice CSE-induced C2C12 myotubes	increased PGC-1α expression by activating SIRT1 deacetylation; alleviated oxidative stress	Deng et al. (2023)
	Resveratrol	50 mg/kg	CS + LPS induced COPD rats	increased SIRT1 and PGC-la expression; inhibited inflammation	Wang et al. (2017b)
	Hesperidin	25,50 mg/kg	CSE-induced COPD mice	activated the SIRT1/PGC-1α signaling pathway; alleviated inflammation and oxidative stress	Wang et al. (2020b)
	AICAR	25 mg/kg (*in vivo*) 2.5 ng/mL *(in vitro)*	CS + *Klebsiella* pneumonia suspension induced COPD rats; L6 myoblasts	upregulated PGC-α by activating AMPK-α; increased levels of MMP and ATP	Mao et al. (2020)
	Aminophylline	2.7 mg/kg	CS + *Klebsiella* pneumonia suspension induced COPD rats	upregulated PGC-α by activating AMPK-α; increased levels of MMP and ATP	Mao et al. (2020)
	Aminophylline	10,20 μg ⁄ml	HPBE cells	activated the CREB-PGC-1α signaling pathway; increased the mtDNA/nDNA ratio and ATP production	Wei et al. (2019)

### 6.1 Targeting mtROS

The therapeutic strategies targeting mtROS have recently become popular in the field of biomedical research. Enhancing mitochondrial antioxidant capacity by supplementing with mitochondrial-targeted antioxidants, such as Mitoquinone (MitoQ), MitoTEMPO, and SS-31, alleviates mitochondrial dysfunction (Gao et al., 2022; Hara et al., 2013; Ahmad et al., 2015). Currently, various mtROS-targeted therapeutic strategies have demonstrated potential in preclinicalCOPD studies. MitoQ, a mitochondrial-targeted antioxidant, consists of CoQ10 and a triphenylphosphine (TPP) cation. CoQ10 is a non‐prescription nutritional supplement found in all tissues and organs in the body. CoQ10 is formed mainly in the inner membrane of mitochondria, where it acts as an electron carrier and participates in the “tricarboxylic acid cycle” to produce ATP by transferring and transporting electrons (Hargreaves, 2021). CoQ10 also serves as a potent lipid-soluble antioxidant,and its antioxidant effects help protect mitochondria from oxidative stress damage, thereby maintaining their normal function (Hargreaves, 2003). The high lipophilicity and stable cationic properties of TPP enable it to easily cross biological membranes and accumulate in the mitochondria, improving its ability to prevent and treat mitochondrial oxidative damage hundreds of times compared to traditional antioxidants (Jelinek et al., 2018; Hu et al., 2021; Gottwald et al., 2018). Traditional antioxidants may exhibit beneficial effects in reducing oxidative damage, but they have not achieved ideal clinical results, largely because they are not effectively absorbed by mitochondria. However, MitoQ can overcome this limitation by terminating mitochondrial lipid peroxidation and reducing the damaging effect of mtROS on mitochondrial/cellular components, thereby preventing mitochondrial dysfunction. (Zhang et al., 2019). Damage to the pulmonary vascular endothelial barrier plays a vital role in CSE-induced COPD pathology. Mitochondrial damage has been reported as a key factor in CSE-induced dysfunction of the pulmonary vascular endothelial barrier; however, MitoQ reverses this process by suppressing ROS production and excessive autophagy, thus reducing mitochondrial damage and CSE-induced human umbilical vein endothelial cells (HUVECs) injury (Chen et al., 2019). Wiegman et al. found that ozone exposure induced airway hyper-reactivity and lung inflammation in mice, accompanied by mitochondrial dysfunction, such as increased mtROS levels and reduced ATP in the lungs; however, these pathological changes were substantially reversed using MitoQ (Wiegman et al., 2015b). *In vitro* experiments revealed that airway smooth muscle (ASM) cells from patients with COPD also exhibited characteristics of mitochondrial dysfunction and the proliferation of ASM cells was inhibited when MitoQ was administered (Wiegman et al., 2015b). Furthermore, despite being a well-known antioxidant, NAC did not considerably affect the MDA concentration or superoxide dismutase 2 (SOD2) gene expression in ozone-exposed COPD mice (Li F. et al., 2013). However, mitochondria-targeted antioxidants such as MitoQ appear to be more effective than traditional antioxidants (Wiegman et al., 2015b; Li F. et al., 2013). This discovery suggests new therapeutic strategies that emphasise the importance of targeting mtROS for the treatment of COPD.

MitoTEMPO, a mitochondrial antioxidant, can directly target mitochondria through the targeted design of antioxidants and related carriers, and can be rapidly transformed into a ubiquinol form with strong antioxidant activity, effectively eliminating ROS and suppressing oxidative stress (Ding et al., 2017). In a CSE-induced cellular senescence model, HFL1 cells treated with CSE exhibited increased mtROS levels, decreased ATP, and impaired mitophagy; however, MitoTEMPO treatment significantly reduced mtROS, decreased perinuclear accumulation of damaged mitochondria, and inhibited cellular DNA damage, partially delaying cellular senescence (Ahmad et al., 2015). This provides a promising therapeutic approach for treating the cellular senescence mechanism in COPD. Moreover, in the COPD cell model, HBECs exposed to CSE exhibited mitochondrial fragmentation and a considerable decrease in MMP. However, MitoTEMPO partially inhibited CSE-induced mitochondrial fragmentation by reducing mtROS levels, effectively delaying HBECs senescence (Hara et al., 2013).

SS-31 is a mitochondria-targeted peptide that selectively localizes to the inner mitochondrial membrane and reduces mtROS accumulation in a dose-dependent manner (Cai et al., 2015). Although the exact mechanism of SS-31 absorption by mitochondria is unclear, it likely independent of the mitochondrial potential (Zhao et al., 2004). Following stimulation of 16HBECs with BRPM2.5, mtROS levels substantially increased; however, SS-31 treatment effectively lowered mtROS levels, promoted ATP production, and suppressed inflammatory response (Gao et al., 2022). Thus, SS-31 has the potential to be an effective treatment strategy for targeting mitochondria to improve oxidative stress.

Several traditional antioxidants have difficulty penetrating the cell and mitochondrial membranes, limiting their application in mitochondrial protection. Mitochondria-targeted antioxidants, which act directly on mitochondria, aim to restore mitochondrial efficiency and show superior effects compared with traditional antioxidants (Wiegman et al., 2015b; Li F. et al., 2013). Therefore, developing novel mitochondria-targeted antioxidants for clinical applications is an urgent task in modern pharmacology.

### 6.2 Targeting mitophagy

Regulation of mitophagy or cellular autophagy is another attractive therapeutic strategy for treating COPD. Melatonin, an important hormone secreted by the pineal gland, has strong antioxidant, anti-inflammatory, and mitochondrial protective abilities, and its lipophilicity allows melatonin to easily cross cell membranes and reach subcellular compartments to exert its effects (Barlow et al., 2019). Melatonin has substantial therapeutic potential as a modulator of mitophagy in various diseases, including anoxia/reoxygenation injury (Bai et al., 2021), renal fibrosis (Yoon et al., 2021), and white matter damage (Qin et al., 2021). Melatonin effectively alleviates cellular damage and pathological progression in these diseases by regulating mitophagy. In a COPD mouse model, CS exposure led to endoplasmic reticulum stress, mitochondrial damage, and increased NLRP3 inflammasome formation; however, melatonin successfully reversed these unfavourable changes through its antioxidant properties and ability to modulate mitophagy, contributing to the restoration of CS-induced lung pathological changes (Mahalanobish et al., 2020).

SIRT1 is an NAD (+)-dependent protein/histone deacetylase that is widely involved in physiological and pathological processes, such as apoptosis (Ke et al., 2023), aging (Wu et al., 2022), inflammation (Zhou et al., 2021), and oxidative stress (Rada et al., 2018). Recently, SIRT1 role in autophagy has received widespread attention; it can induce autophagy in diseases, such as cerebral ischemia/reperfusion injury (Xie et al., 2022), systemic lupus erythematosus (Ke et al., 2022), and osteoporosis (Yang et al., 2019). Its expression is reduced in the lungs of patients with COPD, and its activation may be an effective strategy for treating COPD (Yao et al., 2012). In a CS-exposed COPD mouse model, SIRT1 deficiency led to increased acetylation levels of Forkhead Box O3(FOXO3), decreased PINK1 protein levels, and weakened mitophagy, which further exacerbated cellular senescence and airway resistance. However, these unfavourable changes were reversed after treatment with a SIRT1 activator SRT1720, effectively reducing cell apoptosis and oxidative stress (Jiang et al., 2022).

pirfenidone, a classical drug used to treat idiopathic pulmonary fibrosis, can improve COPD by regulating mitophagy (Araya et al., 2019). In a CS-induced COPD model, PRKN-knockout mice exhibited more substantial airway wall thickening and emphysema than wild-type mice, along with mitochondrial damage and the accumulation of senescent cells, indicating that PRKN plays an important role in regulating mitochondrial function and cell senescence (Araya et al., 2019). PRKN overexpression induced mitophagy in HBECs, effectively reducing mtROS production and delaying cell senescence. Subsequent intervention with pirfenidone in HBECs revealed that pirfenidone induced PRKN protein expression, thereby enhancing mitophagy and preventing the accumulation of mtROS and cell senescence caused by CSE exposure (Araya et al., 2019). This finding proves that pirfenidone is a novel strategy for the treatment of COPD, especially for delaying cell senescence.

Quercetin, a natural flavonoid compound mostly present in the form of glycosides, is commonly found in vegetables, fruits and Chinese herbal medicines, including *Scutellaria baicalensis, Radix glehniae, Semen coicis*, etc. Its activity is reflected in antioxidation (Qi et al., 2022), anti-inflammatory (Yuan et al., 2020), anti-tumor (Murata et al., 2023), immune regulation (Muthian and Bright, 2004), etc., and it can effectively treat respiratory diseases (Xu et al., 2024),allergic diseases (Jafarinia et al., 2020), autoimmune diseases (Shen et al., 2021), cardiovascular diseases (Zhang W. et al., 2023), etc. In a randomized, double-blind, placebo-controlled trial, oral quercetin-containing supplements (200 mg quercetin) administered for 4 weeks effectively alleviated allergy symptoms caused by pollinosis, without any adverse events reported (Yamada et al., 2022). Due to its extensive pharmacological activities, quercetin exhibits therapeutic potential for COPD. It not only improves airway epithelial regeneration by increasing genes expression involved in the epithelial development/differentiation of COPD (McCluskey et al., 2023), but also ameliorates emphysema caused by exposure to CS in mice through antioxidant and anti-inflammatory effects (Araújo et al., 2022). A clinical trial involving subjects with COPD demonstrated good safety and tolerability after taking quercetin (Han et al., 2020). It is also effective in regulating mitophagy (Son et al., 2018). Compared to untreated cells, p-Drp1 and PINK1 expressions in BEAS-2B cells treated with CSE increased by 14.9 times and 6.7 times respectively. Quercetin treatment reduced mitophagy regulatory proteins expression, prevented cell viability decrease caused by CSE exposure, and inhibited mitochondrial dysfunction by increasing functional mitochondria and reducing mtROS production (Son et al., 2018).

Thymoquinone (TQ), a bioactive benzoquinone compound mainly found in Nigella sativa seed oil, has therapeutic effects on cancer (Imran et al., 2018), neurodegenerative diseases (Mahmud et al., 2022), and autoimmune diseases (Ali et al., 2021). Its bioactivities mainly manifest in antioxidant (Isaev et al., 2023), anti-inflammatory (Akter et al., 2021), immunomodulatory (Ali et al., 2021), and anti-tumor (Ahmad et al., 2019) effects. However, due to its poor bioavailability and low oral absorption rate, there are few clinical trials using TQ alone. Targeted delivery of TQ combined with biological nanomaterials can achieve better therapeutic effects than TQ alone (Liu et al., 2022). TQ considerably inhibited lung inflammation caused by CS exposure. In a COPD rat model with 3 months of continuous CS exposure, an appropriate dose of TQ had anti-inflammatory and anti-apoptotic effects (Yetkin et al., 2020). However, long-term TQ administration may cause cumulative toxic effects (Yetkin et al., 2020). Following CSE induction, BEAS-2B cells viability decreased, PINK1 and p-DRP levels significantly increased; however, pretreatment with TQ improved cell viability and regulated mitophagy by reducing PINK1 and p-DRP expression, thereby exerting a protective effect on BEAS-2B cells (Dera et al., 2020).

Puerarin is an isoflavone derivative isolated from pueraria lobata. Puerarin has the functions of regulating intestinal microbiota (Yang et al., 2022), protecting cardiomyocytes (Ding et al., 2023), lowering blood sugar (Li Z. et al., 2022), inhibiting ferroptosis (Ding et al., 2023), and anti-inflammatory (Jeon et al., 2020) effects. Currently, three main dosage forms of puerarin, namely, injection, eye drops, and lyophilized powder, have been approved by the State Food and Drug Administration in China and are clinically used to treat cardiovascular diseases, cerebral infarction, glaucoma (Liu et al., 2023). A novel type of puerarin nanosuspension exhibits high anti-cancer activity *in vitro* and *in vivo* experimentsand low toxicity (Wang et al., 2013). Puerarin can effectively reverse the inflammatory response and oxidative stress of mouse lung tissue caused by acute smoking exposure, thus exerting a significant protective effect on lung tissue (Zhang P. et al., 2022). In HBECs induced by 20% CSE, ROS levels increased significantly, ATP content decreased, and PINK1 and Parkin proteins increased, indicating that mitochondrial function was impaired, leading to excessive activation of mitophagy; however, puerarin reversed these changes, and its protective effect increased with increasing dose, with 200 μg/mL showing good therapeutic effect (Wang L. et al., 2022). This discovery provides strong support for the application of puerarin in respiratory diseases.

In addition to the regulation of mitophagy, autophagy in airway epithelial cells has received considerable attention. A commonly used autophagy inhibitor, 3-Methyladenine (3-MA), blocks autophagosome formation by inhibiting class III phosphoinositide 3-Kinase (PI3K) activity, thereby suppressing autophagy (Shi et al., 2012). Huang et al. reported that in a COPD mouse model, the expression of the autophagic marker, LC3B, was significantly increased in the airway epithelium and lung tissue (Huang et al., 2021). This enhanced autophagy exacerbated bronchial inflammatory changes; however, 3-MA considerably reduced the bronchial inflammatory response in COPD mice (Huang et al., 2021). Similarly, Liu et al. demonstrated that CS exposure induced autophagosome formation and upregulated LC3B expression in airway epithelial cells in a COPD mouse model, leading to airway inflammation and airway remodelling; however, the application of 3-MA effectively alleviated these pathological changes by inhibiting autophagy (Liu et al., 2021). The 3-MA has a protective effect against COPD. However, activating autophagy reduced CSE-induced endothelial cell apoptosis, and 3-MA, which inhibits autophagy, exacerbated CSE-induced cell apoptosis, leading to the development of COPD (Zong et al., 2021). Although 3-MA,can effectively suppress autophagy, there are conflicting reports on its effect on COPD, which partly depend on the controversy surrounding the role of mitophagy in COPD pathogenesis. Currently, the exact mechanism and role of mitophagy in COPD remain unclear and require further exploration.

### 6.3 Targeting mitochondrial dynamics

The inhibition of mitochondrial fragmentation appears to be beneficial in COPD pathogenesis. Mitochondrial Division Inhibitor 1(Mdivi-1), a selective mitochondrial fission inhibitor, improves mitochondrial function by inhibiting Drp1 activity (Su et al., 2023). In a CS-induced COPD mouse model, Mdivi-1 protected airway function (Mizumura et al., 2014). *In vitro*, CSE exposure led to the accumulation of mtROS, induced the phosphorylation of Drp1; however, MitoQ, as an efficient mtROS scavenger, reduced the generation of mtROS and effectively inhibited the phosphorylation of Drp1, indicating that Drp1 is regulated by mtROS. Subsequently, Mdivi-1 treatment significantly inhibited mitochondrial fission in CSE-induced BEAS-2B cells, reversed the decrease of ΔΨm, and reduced cell mortality. Mitochondrial fission, a process regulated by Drp1, plays a crucial role in triggering mitophagy (Son et al., 2018). Mild oxidative stress specifically triggers mitophagy in a Drp1-dependent manner (Frank et al., 2012). This implies that inhibiting Drp1 can suppress mitophagy. Notably, recent research has applied the pharmacological inhibitor of Drp1, Mdivi-1, in studies on mitophagy (Givvimani et al., 2012), demonstrating that Mdivi-1 treatment also inhibits mitophagy in BEAS-2B cells (Mizumura et al., 2014). This provides us with a broader perspective on the biological functions of Mdivi-1 and its applications in the medical field.

Tanshinone IIA is a representative lipid-soluble component found in the traditional Chinese medicine, Danshen. Through sulfonation, it is transformed into the metabolically stable sodium tanshinone IIA sulfonate. Sodium tanshinone IIA sulfonate is effective in treating COPD, which can alleviate inflammatory response and prevent acute exacerbation of COPD by inhibiting the activation of extracellular signal-regulated kinases 1 and 2 (ERK1/2) and nuclear factor kappa-light-chain-enhancer of activated B cells (NF-κB) (Li D. et al., 2020), and improve airway dehydration symptoms by stimulating Cl-secretion in mouse tracheal epithelium (Chen P. X. et al., 2017). It also improves mitochondrial function by regulating mitochondrial dynamics (Guan et al., 2021). The CSE induction on A549 cells resulted in a substantial increase in Drp1 protein and a decrease in Mfn2 protein; however, sodium tanshinone IIA sulfonate treatment alleviated these abnormal changes and prevented the loss of Δψm and inhibited apoptosis (Guan et al., 2021). These findings provide a new mechanism for COPD prevention using sodium tanshinone IIA sulfonate.

Inhibiting mitochondrial fragmentation has multiple effects in COPD, which improves mitochondrial function, alleviates inflammatory response, and reduces cell apoptosis. However, research in this area is still in its initial stages, and more in-depth studies are required to confirm its effectiveness and safety.

### 6.4 Targeting mitochondrial biogenesis

The promotion of mitochondrial biogenesis improves COPD. Given the critical role of PGC-1α in regulating mitochondrial biogenesis, activation of PGC-1α is a potential strategy to enhance mitochondrial biogenesis and improve COPD. SIRT1 mainly activates PGC-1α through deacetylation, while AMPK activates it through phosphorylation; both play vital roles in regulating PGC-1α activity and are crucial components of the PGC-1α upstream signalling pathway (Xue et al., 2019; Wang D. et al., 2022). In a model of COPD with skeletal muscle dysfunction, CSE exposure induced downregulation of PCG-1α expression in C2C12 myotubes, accompanied by pathological changes, such as substantial reduction in myotube diameter and atrophy; however, Glycyl-L-Histidyl-L-Lysine-Copper (GHK-Cu) effectively reversed the downregulation of PCG-1α, increased mitochondrial number in a concentration-dependent manner, enlarged the diameter of myotubes, and alleviated oxidative stress (Deng et al., 2023). The use of the SIRT1-specific inhibitor EX527 abolished the effect of GHK-Cu on PCG-1α expression, indicating that GHK-Cu increased PGC-1α expression by activating SIRT1 deacetylation, thereby promoting mitochondrial biogenesis and improving skeletal muscle dysfunction (Deng et al., 2023).

Resveratrol, a non-flavonoid polyphenolic organic compound abundant in *Veratrum album*, *Polygonum cuspidatum*, grapes, and mulberries, has various pharmacological activities, including antioxidant. (Wang Q. et al., 2022), anti-inflammatory (Mahjabeen et al., 2022), and immune regulation (He et al., 2022). The Food and Drug Administration of the United States has evaluated the safety of resveratrol as Generally Recognized as Safe. This means that under appropriate intake, people generally do not need to worry about the safety of resveratrol. Administration of an RE dosage of 20 mg/kg/day for 28 days to rats showed no adverse effects in the animals (Juan et al., 2002). Although numerous studies have suggested that resveratrol is a well-tolerated and safe compound in humans (Cottart et al., 2010), there are other studies that have reported toxic effects of resveratrol both *in vitro* and *in vivo* (Calabrese et al., 2010). The antioxidant effect of resveratrol is highly recognized. Resveratrol can resist the damage of ROS to DNA, and can effectively inhibit free radicals generation to protect cells from oxidative stress (Shaito et al., 2020). Resveratrol can directly or indirectly activate SIRT1, thereby exerting antioxidant and anti-aging effects. The expression and activity of SIRT1 decrease in aging-related diseases such as COPD, and activating SIRT1 has become an important strategy for treating these diseases. Resveratrol, a known SIRT1 agonist, has been proven to improve mitochondrial function by stimulating SIRT1 expression and maintaining PGC-1α activity in metabolic diseases (Lagouge et al., 2006), COPD (Wang X. L. et al., 2017). COPD rats exposed to CS + Lipopolysaccharide (LPS) exhibited severe inflammatory cell infiltration, alveolar wall thickening, and enlarged alveolar sacs, which were alleviated by resveratrol treatment (Wang X. L. et al., 2017). CS + LPS exposure not only increased IL-6 and IL-8 levels but also reduced SIRT1 and PGC-1α expression; however, resveratrol inhibited inflammation while enhancing SIRT1 and PGC-1α expression, suggesting that the effect of resveratrol on airway inflammation induced by CS + LPS exposure may be mediated via the SIRT1/PGC-1α pathway (Wang X. L. et al., 2017). Another study also showed after mice inhaled resveratrol continuously for 3 months, the decline in lung compliance was significantly delayed. During this process, resveratrol was able to activate SIRT1 and maintain PGC-1α level, effectively slowing down lung function deterioration by preserving alveolar epithelial type 2 cells integrity (Navarro et al., 2017). These findings are in stark contrast to the observations made in experimental animal models,suggesting complex and partially understood mechanisms in the translation from animal models to human diseases. Although the therapeutic effect of resveratrol in treating COPD through activating SIRT1 and PGC-1α expression have been verified in animal models, a double-blind, randomized, placebo-controlled study revealed contrasting results (Beijers et al., 2020). In this study, COPD patients who received resveratrol treatment (150 mg/day) for 4 weeks did not experience significant improvement in skeletal muscle mitochondrial function. Moreover, compared to the placebo group, no significant differences of SIRT1 and PGC-1α expression in the resveratrol group (Beijers et al., 2020).

Hesperidin, a flavanone glycoside widely found in the peel of citrus fruits, has various pharmacological activities, such as antioxidant (Imperatrice et al., 2022), anti-inflammatory (Adefegha et al., 2021), cell senescence delay (Han et al., 2023), and apoptosis inhibition (Aboraya et al., 2022). In a COPD mouse model, hesperidin was shown to reverse pathological changes in the lung tissue, such as inflammatory cell infiltration, small airway thickening, and alveolar septal collapse. By activating the SIRT1/PGC-1α signalling pathway, hesperidin alleviated inflammation and oxidative stress induced by CSE injection in mice, with higher concentrations showing superior efficacy compared with that at lower concentrations (Wang S. et al., 2020).

AMPK activators, such as metformin (Nakashima et al., 2022), 5-Aminoimidazole-4-carboxamide ribonucleotide (AICAR) (Mao et al., 2020), aminophylline (Mao et al., 2020), quercetin (Mitani et al., 2017), and resveratrol (Qi et al., 2014) ameliorate COPD by activating AMPK. In the COPD rat model, AICAR upregulated PGC-α by activating AMPK-α in skeletal muscle, thereby promoting mitochondrial biogenesis and improving skeletal muscle dysfunction in COPD rats (Mao et al., 2020). Aminophylline administration also resulted in this positive effect (Mao et al., 2020).

In addition to the known key factors, SIRT1 and AMPK, that regulate PGC-1α, cAMP Response Element-Binding Protein (CREB), an upstream signal, promotes mitochondrial biogenesis by activating PGC-1α, which has been verified in bronchial asthma (Wang H. et al., 2019), neurodegenerative diseases (Yang et al., 2020), and diabetic nephropathy (Chen et al., 2022). As a non-selective phosphodiesterase inhibitor, aminophylline, exhibited pharmacological effects in promoting mitochondrial biogenesis to improve mitochondrial function (Wei et al., 2019). In HPBECs, aminophylline promoted mitochondrial biogenesis by activating the CREB-PGC-1α signalling pathway, and its impact was reflected in the increase of the mtDNA/nDNA ratio, elevation of mitochondrial protein cytochrome B, and enhancement of mitochondrial respiratory rate and ATP production (Wei et al., 2019). Notably, when the specific inhibitor H89 was used to inhibit the activation of CREB, the inductive effect of aminophylline on PGC-1α and its influence on the mtDNA/nDNA ratio and respiratory rate were eliminated, further demonstrating that the pharmacological effects of aminophylline relied on the activation of CREB (Wei et al., 2019).

In summary, the promotion of mitochondrial biogenesis may positively affect COPD treatment. However, this is not a direct strategy to treat COPD. Effective promotion of the synergistic effect of mitochondrial biogenesis with existing therapies requires further exploration.

## 7 Conclusions and future prospects

Despite progress in understanding mitochondrial quality control in COPD and the therapeutic potential of various mitochondria-targeted drugs, some issues remain to be addressed. Firstly, mitochondrial quality control involves multiple complex biological processes and signalling pathways, and its mechanisms are not completely understood. Although some key mechanisms have been identified, such as mitophagy, mitochondrial dynamics, and mitochondrial biogenesis, their interactions and regulatory networks require further exploration. Mitochondrial function varies across different cell types and physiological and pathological conditions. Therefore, a comprehensive understanding of the mitochondrial quality control mechanism requires further investigation under diverse conditions. Additionally, the mechanism is a hierarchical network where disruptions in one aspect, such as mitochondrial dynamics, can impact the overall morphology, structure and function of mitochondria, leading to various diseases.

Secondly, translating mitochondria-targeted drugs to clinical use remains challenging. The central role of mitochondria in cellular function and various physiological processes means that disturbances in their function can have widespread effects. Variations in mitochondria-targeted drug dosage and timing may lead to side effects, such as oxidative stress, fluctuations in energy metabolism, and uncontrolled apoptosis. Therefore, evaluating safety and tolerability is crucial in clinical trials. Furthermore, mitochondria’s double membrane with selective permeability complicates the effective penetration of drugs through the mitochondrial membrane to reach their therapeutic targets. Hence, drug design must consider molecular size, electric charge, and hydrophilic/hydrophobic properties to ensure passage through the mitochondrial membrane.

Thirdly, the mechanisms and extent of mitochondrial damage may vary in different diseases. Currently, eliable biomarkers lack, which accurately assess the extent of mitochondrial damage and drug efficacy, impeding the objective measurement of drug responses in clinical trials. However, disruptions in mitochondrial quality control are crucial in COPD onset and progression, regardless of aetiology. Mitochondria-targeted therapies show promise in COPD models. Therefore, understanding the link between mitochondrial quality control and COPD is crucial for revealing its pathogenesis, discovering novel therapeutic targets, and developing personalised therapeutic strategies. Future research should offer novel insights into COPD pathology and lay the foundation for mitochondria-targeted drug development. Meanwhile, the research and application of mitochondria-targeted drugs remain an intricate domain for further advancements and innovations.

## Glossary

COPD: Chronic obstructive pulmonary disease
ROS: Reactive oxygen species
OXPHOS: Oxidative phosphorylation
mtROS: Mitochondrial ROS
PGC1-α: Peroxisome Proliferator-Activated Receptor Gamma Coactivator 1-Alpha
CNKI: China National Knowledge Infrastructure
MMP: Mitochondrial membrane potential
NAD: Nicotinamide Adenine Dinucleotide
FAD: Flavin Adenine Dinucleotide
ETC: Electron transport chain
CoQ10: Coenzyme Q10
Cytochrome c: Cytochrome c Oxidase
HIFs: hypoxia-inducible factors
PKB/AKT: Protein Kinase B
PTEN: Phosphatase and Tensin Homolog
RIPK1: Receptor-Interacting Protein Kinase 1
RIPK3: Receptor-Interacting Protein Kinase 3
AMPK: AMP-activated Protein Kinase
HIF-1α: Hypoxia-Inducible Factor 1-Alpha
mTOR: Mammalian Target of Rapamycin
SOD: Superoxide dismutase
CAT: Catalase
GPX: Glutathione peroxidase
MnSOD: Manganese superoxide dismutase
FAO: Fatty acid oxidation
CPT1A: Carnitine Palmitoyltransferase 1A
Drp1: Dynamin-Related Protein 1
Ser616: Serine 616
PINK1: PTEN-Induced Kinase 1
MitoTEMPO: Mitochondria-Targeted Tempo
LPO: Lactoperoxidase
TFAM: Mitochondrial transcription factor A
cGAS: Cyclic GMP-AMP Synthase
STING: Stimulator of Interferon Genes
NLRP3: NOD-like Receptor Pyrin Domain Containing 3
MT-ATP6: Mitochondrially Encoded ATP Synthase Membrane Subunit 6
nDNA: nuclear DNA
TGF-β1: Transforming Growth Factor Beta 1
Ub: Ubiquitin
LC3: Microtubule-associated protein 1A/1B-light chain 3
NIX: Nip3-like protein X
BNIP3L: BCL2-interacting protein 3 like
BNIP3: BCL2-interacting protein 3
FUNDC1: FUN14 domain containing 1
BH3: Bcl-2 Homology 3
Atg8: Autophagy-related protein 8
March5: Membrane Associated Ring-CH-Type Finger 5
Mfn: Mitofusin
Opa1: Optic Atrophy 1
INF2: Inverted Formin 2
Spire1C: Spire-type Actin Nucleation Factor 1C
MID49: Mitochondrial Dynamics Protein 49
MIEF1: Mitochondrial Elongation Factor 1
PGC-1α: Peroxisome Proliferator-Activated Receptor Gamma Coactivator 1-Alpha
SIRT1: Sirtuin-1
PRMT1: Protein Arginine Methyltransferase 1
PKA: protein kinase A
TIM23: Translocase of the Inner Membrane 23
FNIP1: Folliculin Interacting Protein 1
FLCN: Folliculin
TFEB: Transcription Factor EB
CS: Cigarette Smoke
CSE: Cigarette Smoke Extract
PKM2: Pyruvate Kinase M2
NEAT1: Nuclear Enriched Abundant Transcript 1
NOX4: NADPH Oxidase 4
Nrf2: Nuclear Factor Erythroid 2-Related Factor 2
PRKN: parkin RBR E3 ubiquitin protein ligase
HFL1: Human Fetal Lung Fibroblast 1
HBECs: Human Bronchial Epithelial Cells
IL-6: Interleukin-6
TNF-α: Tumor Necrosis Factor-Alpha
USP19: Ubiquitin-Specific Peptidase 19
Fis1: Fission 1
16HBE: 16 Human Bronchial Epithelial
pDRP1: Phosphorylated Dynamin-Related Protein 1
ATII: Alveolar Type II Cells
NAC: N-acetylcysteine
MLE12: Mouse Lung Epithelial 12
CIHH: Chronic Intermittent Hypoxia-Hypercapnia
TPP: Triphenylphosphine
MitoQ: Mitoquinone
HUVECs: Human Umbilical Vein Endothelial Cells
ASM: Airway Smooth Muscle
SOD2: Superoxide Dismutase 2
FOXO3: Forkhead Box O3
TQ: Thymoquinone
3-MA: 3-Methyladenine
PI3K: Phosphoinositide 3-Kinase
Mdivi-1: Mitochondrial Division Inhibitor 1
ERK1/2: Extracellular Signal-Regulated Kinases 1 and 2
NF-κB: Nuclear Factor kappa-light-chain-enhancer of activated B cells
GHK-Cu: Glycyl-L-Histidyl-L-Lysine-Copper
LPS: Lipopolysaccharide
AICAR: 5-Aminoimidazole-4-carboxamide ribonucleotide
CREB: cAMP Response Element-Binding Protein
